# Enhancer activation by FGF signalling during otic induction

**DOI:** 10.1016/j.ydbio.2019.09.006

**Published:** 2020-01-01

**Authors:** Monica Tambalo, Maryam Anwar, Mohi Ahmed, Andrea Streit

**Affiliations:** Centre for Craniofacial and Regenerative Biology, Faculty of Dental, Oral and Craniofacial Sciences, King’s College London, London, SE1 9RT, UK

**Keywords:** Cranial ganglia, Cis-regulatory elements, Ear, Gene networks, Hearing, Placode

## Abstract

Vertebrate ear progenitors are induced by fibroblast growth factor signalling, however the molecular mechanisms leading to the coordinate activation of downstream targets are yet to be discovered. The ear, like other sensory placodes, arises from the pre-placodal region at the border of the neural plate. Using a multiplex NanoString approach, we determined the response of these progenitors to FGF signalling by examining the changes of more than 200 transcripts that define the otic and other placodes, neural crest and neural plate territories. This analysis identifies new direct and indirect FGF targets during otic induction. Investigating changes in histone marks by ChIP-seq reveals that FGF exposure of pre-placodal cells leads to rapid deposition of active chromatin marks H3K27ac near FGF-response genes, while H3K27ac is depleted in the vicinity of non-otic genes. Genomic regions that gain H3K27ac act as cis-regulatory elements controlling otic gene expression in time and space and define a unique transcription factor signature likely to control their activity. Finally, we show that in response to FGF signalling the transcription factor dimer AP1 recruits the histone acetyl transferase p300 to selected otic enhancers. Thus, during ear induction FGF signalling modifies the chromatin landscape to promote enhancer activation and chromatin accessibility.

## Highlights

• New direct and indirect FGF targets during otic-epibranchial progenitor induction.
• Identification and *in vivo* activity of enhancers controlling gene expression in otic-epibranchial progenitors.
• Enhancer analysis reveals complex regulation of the transcription factor *Foxi3*.
• FGF signalling enhances H3K27 acetylation at enhancers associated to otic-epibranchial specific genes.
• Downstream of FGF signalling AP1 recruits p300 to otic-epibranchial enhancers.

## 1 Introduction

The vertebrate inner ear is critical to relay auditory and vestibular information from the environment to the brain. Developmental malformations or postnatal damage of inner ear cells lead to permanent sensory defects as the ear does not have the ability to regenerate or repair. Despite extensive studies, our understanding of normal ear development has not yet translated into new biological approaches to alleviate such disorders or to promote sensory cell regeneration *in vivo*. This is partly due to the lack of mechanistic information downstream of signalling pathways that control ear development and of how signalling events are translated into changes in gene expression and cell behaviour.

The Fibroblast Growth Factor (FGF) pathway is crucially important for many steps in ear formation. During early development, FGF signalling mediates the induction of otic-epibranchial progenitors (OEPs) (Ladher et al., 2000; Maroon et al., 2002; Park and Saint-Jeannet, 2008; Phillips et al., 2001; Sun et al., 2007; Urness et al., 2010; Wright and Mansour, 2003; Yang et al., 2013a) from the pre-placodal region, a pool of progenitors that can give rise to other placodes, neural, neural crest and epidermal cells and is located at the border of the anterior neural plate (Saint-Jeannet and Moody, 2014; Schlosser, 2014; Streit, 2008, 2018). Otic precursors segregate from epibranchial progenitors to form a transient structure, the otic placode, and subsequently the otic vesicle, which gives rise to the entire inner ear including the cochlear-vestibular ganglion (Chen and Streit, 2013; Groves and Fekete, 2012; Ladher, 2017; Ohyama et al., 2007; Whitfield, 2015). During these stages, FGF signalling promotes otic neurogenesis and mediates patterning events that subdivide the otic vesicle into distinct functional domains (Abello et al., 2010; Adamska et al., 2001; Alsina et al., 2004; Alvarez et al., 2003; Hammond and Whitfield, 2011; Leger and Brand, 2002; Maier and Whitfield, 2014). As the vesicle gradually acquires the shape of the adult ear, sensory patches emerge that generate auditory and vestibular sensory hair cells and their surrounding supporting cells. Within the cochlea, these cells form a stereotypical pattern that is critical for normal hearing, with FGF signalling controlling both their arrangement and their differentiation (Doetzlhofer et al., 2009; Haque et al., 2016; Mueller et al., 2002; Shim et al., 2005). Finally, FGFs have also been implicated in hair cell survival in adult ears as well as controlling their regeneration from supporting cells (Jiang et al., 2018; Kniss et al., 2016; Lee et al., 2016; Lush et al., 2019).

Upon ligand binding FGF receptors initiate different intracellular cascades including the ERK/MAPK pathway (Ornitz and Itoh, 2015) to regulate gene expression and subsequent changes in cell behaviour. Signalling information is integrated by non-coding regulatory enhancer regions to activate or repress downstream targets (Banerji et al., 1981; Catarino and Stark, 2018; Long et al., 2016; Shlyueva et al., 2014). Active enhancers are characterised by low nucleosome density (Boyle et al., 2008), recruitment of the histone acetylase p300 (Visel et al., 2009), acetylation of histone 3 lysine-27 (H3K27ac) in the enhancer-flanking nucleosomes (Creyghton et al., 2010; Kharchenko et al., 2011; Rada-Iglesias et al., 2011, 2012; Zentner et al., 2011), and the expression of RNA transcripts (Kim et al., 2010; Wang et al., 2011). Downstream of FGF signalling, different mechanisms regulate target gene expression. On one hand, Erk1/2 MAP-kinases can directly modify nucleosome forming histones and change epigenetic marks (Chen et al., 2007; Soloaga et al., 2003), and their inhibition can increase chromatin accessibility (Patel et al., 2013; Semprich et al., 2019). On the other hand, MAP kinases phosphorylate Ets transcription factors (e.g. Etv4 and Etv5) or the dimer forming leucine zippers cFos and cJun (AP1) (Gruda et al., 1994; Neuberg et al., 1989; Ornitz and Itoh, 2015; Tsang and Dawid, 2004; for review Yang et al., 2003, 2013b), which then bind to enhancers and together with other factors, activate target genes. For example, AP1 cooperates with cell type specific transcription factors to recruit the BAF complex, which in turn remodels nucleosomes to establish a permissive chromatin state (Vierbuchen et al., 2017). Thus, modulation of FGF signalling appears to control changes in the epigenetic signature. However, in the context of *in vivo* ear development, the molecular mechanisms that translate FGF signalling into rapid transcriptional changes remain to be elucidated.

Here we identify direct and indirect FGF target genes during the earliest step of ear development, the induction of otic-epibranchial progenitors, by examining changes in expression of more than 200 transcripts that define different cell populations in the embryonic ectoderm. Investigating chromatin changes in response to FGF signalling, we find that FGF stimulation of pre-placodal cells leads to deposition of H3K27ac marks in the vicinity of ear-specific, FGF-response genes and that these genomic regions act as ear-specific enhancers. Finally, our findings suggest that AP1 may play a key role in this process: upon FGF signalling, AP1 recruits the histone acetylase p300 to some selected ear enhancers, which in turn promotes H3K27 acetylation associated with increased chromatin accessibility and enhancer activation. Together these findings highlight that during ear induction, the initial response to Erk/MAPK signalling directly activates ear-specific enhancers, providing a molecular mechanism for rapid activation of gene expression downstream of FGF. In turn, these observations may impact on a variety of diseases and developmental disorders where FGFs play a major role.

## 2 Results

### 2.1 Identification of direct FGF targets in ear progenitors

**Fig. 1:**
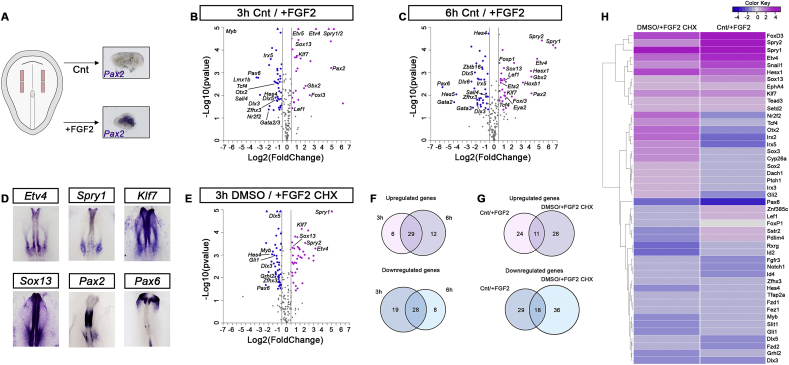
**Identification of direct and indirect FGF targets during OEP induction.** (A) Pre-placodal explants from HH6 chick embryos were cultured in the absence or presence of FGF2. After 6 h, the OEP marker *Pax2* is upregulated. (B) 3 h FGF2 treatment promotes the expression of OEP transcripts, while repressing non-otic and late otic genes as determined by NanoString nCounter. A fold change of 1.5 or 0.25 (grey lines) and a p-value < 0.05 were used as threshold; transcripts not passing these thresholds are shown in grey and significantly up- and downregulated genes are shown in pink and violet, respectively. (C) After 6 h of FGF2 treatment pre-placodal cells continue to express many 3hr-induced transcripts and upregulate new genes. A fold change of 1.5 or 0.25 (grey lines) and a p-value < 0.05 were used as threshold; transcripts not passing these thresholds are shown in grey and significantly up- and downregulated genes are shown in pink and violet, respectively. (D) Genes upregulated by FGF2 are expressed in OEPs, while FGF2-repressed genes are absent from OEPs (*Pax6*) as determined by *in situ* hybridization. (E) Pre-placodal explants were grown in the presence of FGF2 and cycloheximide (CHX) or vehicle control (DMSO) for 3 h. Changes of gene expression was analysed by NanoString. A fold change of 1.5 or 0.25 (grey lines) and a p-value < 0.05 were used as threshold; transcripts not passing these thresholds are shown in grey and significantly up- and downregulated genes are shown in pink and violet, respectively. (F) Venn diagrams showing the overlap between FGF up- or downregulated genes after 3 and 6 h. (G) To identify direct FGF targets in OEPs we compared FGF up- or downregulated genes in controls and CHX-treated explants. (H) Heatmap comparing FGF up- or downregulated genes in controls and in the presence of CHX. Note: transcripts that are induced or repressed in both conditions are considered to be direct FGF targets.

FGF signalling is critical to initiate the ear programme. Loss of FGFs or pathway inhibition results in the complete absence of ear precursors, while exposure of pre-placodal cells to FGF induces otic epibranchial progenitors (OEPs) (Ladher et al., 2000; Maroon et al., 2002; Park and Saint-Jeannet, 2008; Phillips et al., 2001; Sun et al., 2007; Urness et al., 2010; Wright and Mansour, 2003; Yang et al., 2013a). However, FGFs have also been implicated in the induction of olfactory and trigeminal precursors (Bailey et al., 2006; Canning et al., 2008) suggesting that they act in a cell type specific manner. To explore the transcriptional changes in response to FGF on a wide array of downstream targets we used NanoString nCounter as a multiplex approach. Based on recent transcriptome data (Chen et al., 2017) we designed a probe set containing a total of 216 probes including 70 ear specific factors, as well as transcripts normally expressed in progenitors for other sense organs, cranial ganglia, neural and neural crest cells (Supplementary File 1). Pre-placodal cells from HH6 chick embryos were cultured in the absence or presence of FGF2 for 3 and 6 h and processed for NanoString (Fig. 1A). After 3 h known FGF targets (*Etv4/5*, *Spry1/2*) are strongly up-regulated together with early OEP markers (e.g. *Foxi3*, *Gbx2*, *Pax2*, *Sox13* and *Klf7;* in total 16 otic TFs), while genes normally expressed in other cell types (e.g. *Pax6*, *Otx2*, *Msx1*, *Id2*, *-4*) and some late otic genes (*Sall4*, *Zfhx3*, *Fez1*, *Lmx1b, Myb*) are repressed (Fig. 1B, D; Supplementary File 1). In addition to well established OEP genes, this analysis identifies transcripts previously not associated with otic placode induction and/or with FGF regulation such as the chemokine *Cxcl14* (Supplementary Fig. 1), the transcription factors *Klf7*, *Bach2* and *Sox13*, the Wnt receptor *Fzd7* and various chromatin modulators like *Chd7*, *Baza1*, *Setd2* and *Mll2*. 6 hrs after FGF exposure, the expression of most early induced transcripts is maintained and a few new factors become upregulated (e.g. *Eya2*, *N-Myc, Tcf7L2*; Fig. 1C, D, F; Supplementary File 1).

To identify genes regulated by FGF directly, pre-placodal explants were cultured with FGF2 for 3 h in the presence of vehicle control (DMSO) or cycloheximide (CHX) to block protein synthesis. NanoString analysis reveals changes in a total of 83 transcripts (Fig. 1E; 39 up-, 54 downregulated; Supplementary File 1). We restricted our analysis to genes that are normally regulated by FGF signalling in ear progenitors (Fig. 1B) and compared their behaviour in the absence and presence of CHX. We find that of 35 FGF upregulated transcripts (Figure 1B), 11 continue to be induced when protein synthesis is blocked and are therefore considered to be direct targets (Fig. 1G and H). These include genes normally expressed in OEPs like *Spry1* and -*2*, *Etv4*, *Sox13, Klf7*, *Hesx1* and *Tead3*, a neural crest (*FoxD3*) and a mesoderm transcription factor (*Snail1*). Of 47 FGF-inhibited genes, 18 appear to be directly regulated by FGF including *Pax6*, *Dlx3* and -*5*, *Myb*, *Hes4*, *Zfhx3*, *Gli1* and *Grhl2* (Fig. 1G and H).

In summary, 16 TFs normally expressed in otic progenitors are rapidly activated by FGF signalling, of these five are direct targets. At the same time, FGF2 represses genes that characterise anterior placode fates (e.g. lens) as well as neural crest identity.

### 2.2 FGF signalling induces dynamic changes in H3K27 acetylation

**Fig. 2:**
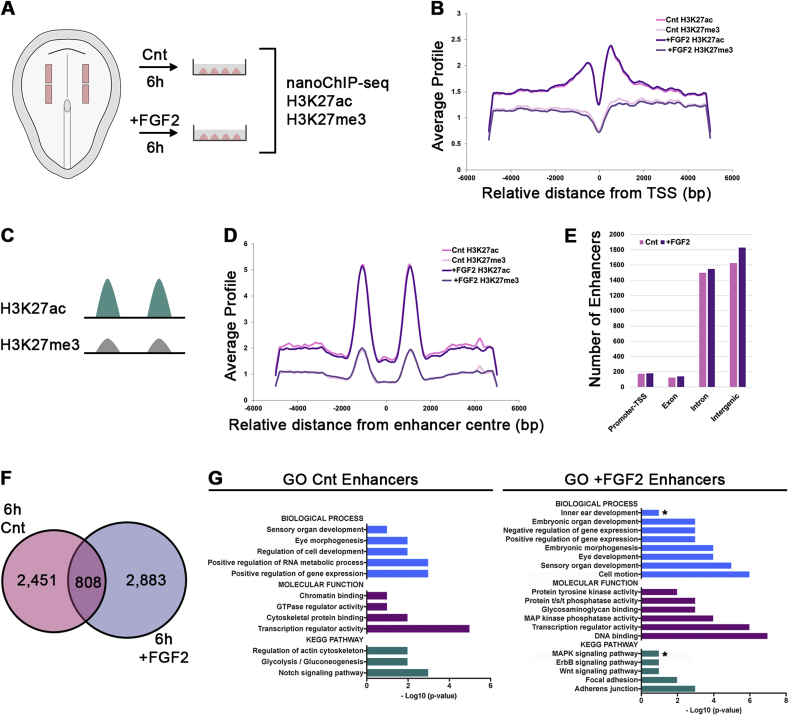
**Identification of FGF-responsive regulatory elements during OEP induction.** (A) Pre-placodal explants were dissected from 0ss (HH6) embryos and cultured in the presence (+FGF2) or absence (Cnt) of FGF2 for 6 ​h. Around 100 tissues per condition were processed for nanoChIP-seq. (B) Average read density of H3K27ac and H3K27me3 in control (pink) and +FGF2 treated cells (violet) around the transcription start site (TSS). Read densities are highest close to the TSS and display a peak-dip-peak pattern where the dip-region may represent bound transcriptional activators or repressors. (C) Bimodal distribution of H3K27ac (green) and absence/low H3K27me3 (grey) was used to identify putative enhancers. (D) In control (pink) and +FGF2 treated explants (violet), the average read density of H3K27ac and H3K27me3 around the centre of putative enhancers exhibit a bimodal distribution. H3K27me3 signals in both control and experimental cells is low, while H3K27ac is high as expected for active enhancers. (E) Bimodal H3K27ac peaks in control (Cnt; pink) and +FGF2 (violet) treated samples show a similar distribution with an around 170 located close to promotors, 130 in exonic, 1500 in intronic and 1700 in intergenic regions. (F) Venn-diagram representing putative enhancer elements unique in control (pink; 2451) and +FGF2-treated pre-placodal cells (violet; 2883). 808 putative enhancers are shared in both conditions. (G) Putative enhancers were associated to the closest TSS. Gene ontology annotation reveals significant enrichment of ear and MAPK signalling related terms (*) in enhancer associated genes in FGF2-treated pre-placodal cells, but not in controls.

Activation of the FGF pathway results in rapid changes of gene expression, and in OEPs this activates the MAP kinase pathway (Yang et al., 2013a). To define the initial events as pre-placodal cells are induced to become OEPs, we assessed changes in Histone 3 lysine 27 acetylation (H3K27ac) and trimethylation (H3K27me3) in response to FGF signalling. Histone acetylation is associated with increased accessibility of chromatin and gene activation, marking active enhancers and promoters, while H3K27me3 is associated with gene repression (for review see: (for review see: Calo and Wysocka, 2013; Kimura, 2013). Pre-placodal cells from HH6 chick embryos were cultured in the presence or absence of FGF2 for 6 h and processed for chromatin immunoprecipitation for H3K27ac and H3K27me3 followed by sequencing (Fig. 2A; Adli and Bernstein, 2011). As expected in both control and FGF-treated cells, transcription start sites (TSS) show H3K27ac accumulation and low levels of H3K27me3 (Fig. 2B). However, genome-wide comparison of both samples reveals significant changes in the distribution of both histone marks.

**Fig. 3:**
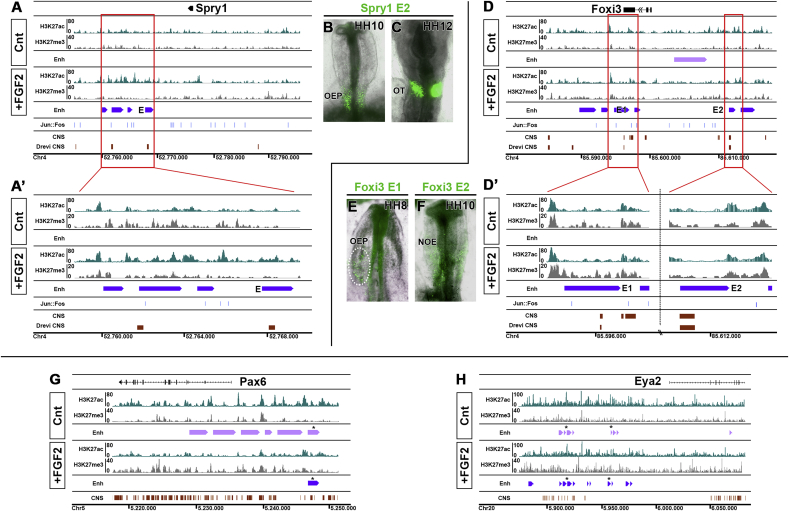
**Ear specific enhancers gain H3K27ac upon FGF stimulation of pre-placodal cells.** IGB browser view of the Spry1 and Foxi3 locus (A, D; close-up; A′, D′), both expressed in OEPs, and of the FGF-repressed gene Pax6 (G) and the sensory progenitor gene Eya2 (H). H3K27ac tracks are shown in green and H3K27me3 tracks in grey. Enh: putative enhancers called by Homer and MACS2 (+FGF2 violet bars; control pink bars). Blue bars: Jun:Fos location of AP1 binding motifs. CNS: conservation from PECAN alignments (ENSEMBL) and DREiVE are shown in red. (B, C) Spry1-E is active in OEPs and the otic cup. (E) *Foxi3-E1* is active in OEPs at the 4–5ss (HH8), while *Foxi3-E2* is activated later at the 10ss (HH10) in the non-otic ectoderm (NOE; F). (G) H3K27ac is lost in the Pax6 locus upon FGF treatment of sensory progenitor cells, while H3K27me3 is increased. The number of putative enhancers is reduced. (H) There are subtle changes in H3K27ac and H3K27me3 in the Eya2 locus. * marks enhancers shared between controls and FGF2-treated cells.

Association of H3K27ac peaks to the nearest TSS reveals that genes normally expressed in OEPs like the direct FGF targets *Spry1* and *Spry2*, the FGF-responsive factors *Foxi3, Hesx1* and *Cxcl14* show a marked gain in H3K27ac (Fig. 3A, A′, D, D’; Supplementary Fig. 2; Supplementary Fig. 3A). In contrast, transcripts normally absent from OEPs and repressed by FGF signalling like *Pax6*, *Dlx5/6* and *Gata3* show a significant reduction but gain trimethylation (Fig. 3G; Supplementary Figs. 3B–C), while there is no change in H3K27ac near pre-placodal genes like *Eya2* (Fig. 3H), whose expression precedes OEP induction by FGF (Ahrens and Schlosser, 2005; Litsiou et al., 2005; Pandur and Moody, 2000; Sato et al., 2010). Thus, changes in histone acetylation reflect changes in gene expression as pre-placodal cells are specified as OEPs.

### 2.3 H3K27ac marks FGF-responsive enhancers in OEPs

Activation of gene expression is controlled by cis-regulatory elements and active enhancers are normally flanked by H3K27ac peaks (Fig. 2C). Therefore, the increase in H3K27ac upon FGF stimulation may indicate the activation of ear enhancers. To identify putative enhancers genome-wide, we extracted bimodal H3K27ac peaks with a maximum distance of 3 kb (Fig. 2D) in pre-placodal cells (control) and FGF-induced OEPs; these are mostly located in intronic or intergenic regions (Fig. 2E). We find 2451 putative enhancers that are unique to control cells, 2883 specific to FGF2-treated progenitors, and 808 are common to both (Fig. 2F, Supplementary Fig. 4). FGF-induced putative enhancers preferentially associate with genes enriched in otic progenitors (from Chen et al., 2017): comparing all OEP transcripts to those with putative active enhancers shows significantly higher expression levels of the latter (Supplementary Fig. 5). In addition, GO term analysis confirms that putative enhancer associated genes are linked to terms like inner ear development and MAP kinase signalling, while these terms are absent from genes in controls (Fig. 2G).

**Fig. 4:**
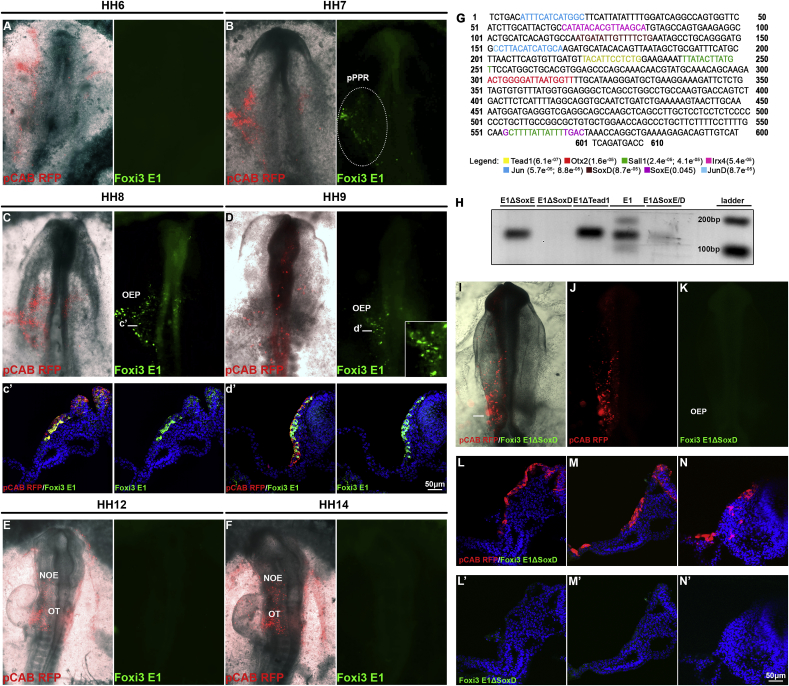
***In vivo* activity of the *Foxi3-E1* enhancer and its transcriptional inputs.** *Foxi3-E1* driving eGPF and b-actin promoter driving RFP (pCAB RFP) were coelectroporated into chick embryos at primitive streak stages, and enhancer activity was monitored from 0 to 1ss onwards. (A, B) A few *Foxi3-E1* eGFP ​+ ​cells were first identified at HH7 in posterior pre-placodal region (pPPR); between HH8-9 the enhancer shows broad activity in OEPs (C, D). (c’, d’) Transverse sections at the level of OEPs show *Foxi3-E1* activity in the ectoderm. To assess whether *Foxi3 E1* continues to be active as *Foxi3* is downregulated in the otic placode embryos were electroporation at 5–6ss. *Foxi3-E1* does not activate eGFP expression at later stages HH12 (E) and HH14 (F). (G) 610bp *Foxi3-E1* sequence. Coloured sequences indicate computationally identified transcription factors binding motifs. RSAT and Clover p-values are reported in the legend. Only factors that are also expressed in otic cells are considered. (H) Deletion of the SoxE, SoxD, Tead1 and SoxE/SoxD binding sites. One-step RT-PCR of OEP dissected tissues electroporated with the mutated constructs shows that SoxD, as well as SoxE/SoxD deletions, abrogates *Foxi3-E1* activity. (I–K) Electroporation of Foxi3-E1ΔSoxD and pCAB RFP plasmids at primitive streak stages confirms that the SoxD binding site is required for *Foxi3-E1* activity: no eGFP reporter activity is (n = 7). (L–N) Representative transverse sections at the level of OEPs of three different embryos show no eGFP-reporter activity in the ectoderm (L′-N′), pointing to SoxD transcription factors (Sox5/6/13) as one upstream input. pPPR: posterior pre-placodal region, OEP: Otic-epibranchial progenitors, NOE: non-otic ectoderm, OT: otic placode.

**Fig. 5:**
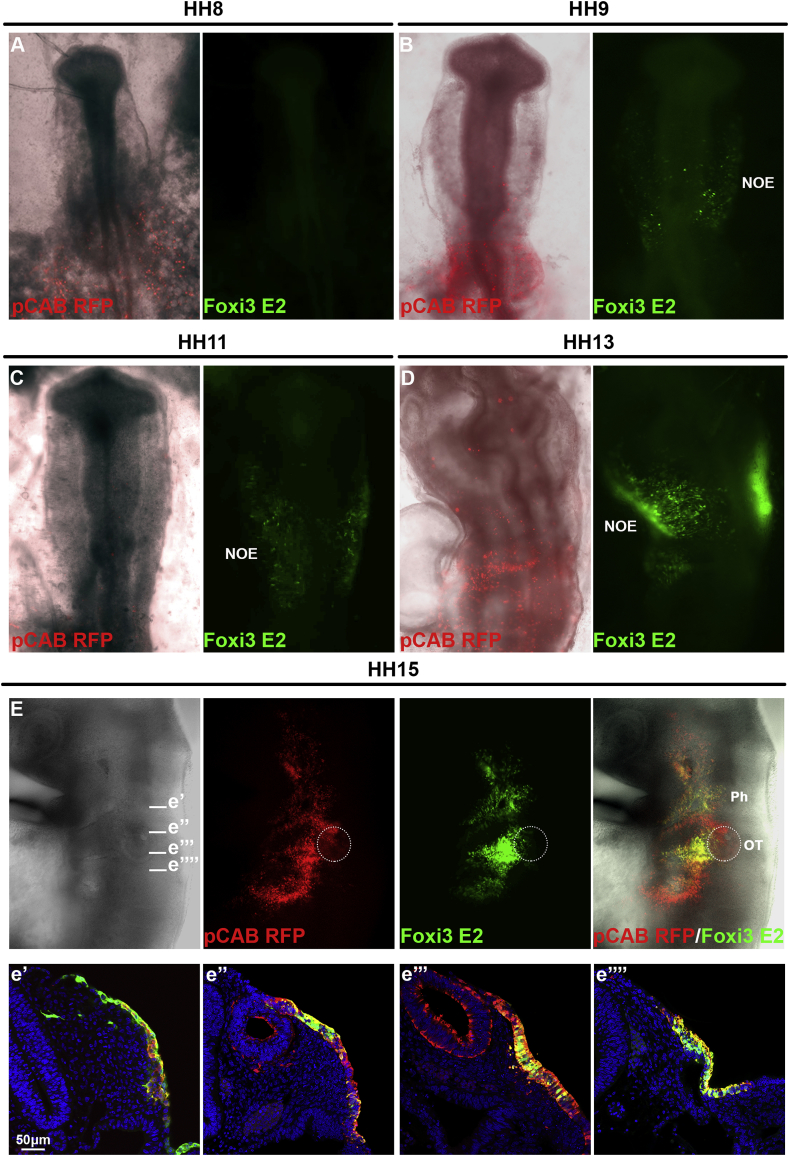
***In vivo* activity of the *Foxi3-E2* enhancer.** *Foxi3-E2* driving eGPF and β-actin promoter driving RFP were coelectroporated into chick embryos at primitive streak and somite stages, and enhancer activity monitored from 0 to 1ss onwards. *Foxi3-E2* eGFP ​+ ​cells were first identified at HH9 in the non-otic ectoderm (NOE) (A, B) and eGFP was maintained at later stages (C, D). At around HH15 the enhancer remains active in the pharyngeal arche ectoderm ventral to the otic placode or vesicle (Ph). The otic vesicle (Ot; circle) has been widely electroporated but no reporter activity is detected (E). Sections anterior and posterior to the otic vesicle show enhancer expression in the non-otic ectoderm (e’-e’’’’). Overall the activity of Foxi3 E2 recapitulates the late expression of *Foxi3*.

To assess whether the genomic regions flanked by H3K27ac are indeed active OEP enhancers *in vivo*, we selected elements associated to known FGF targets or OEP genes, cloned them into reporter constructs to drive eGFP and assessed their activity in chick embryos. HH6 embryos were electroporated with enhancer-eGFP vectors together with a control vector driving RFP ubiquitously. There are four putative enhancers immediately downstream of *Spry1* (Fig. 3A). We determined that *Spry1-E* is conserved across vertebrates as assessed by DREiVE (Khan et al., 2012) (Fig. 3A) and, when tested for *in vivo* activity, drives eGFP in otic progenitors from the 5-somite stage (ss) onwards (Fig. 3B and C) recapitulating the normal expression pattern of *Spry1* (compare to Fig. 1D). In contrast, a putative enhancer element a further away does not show any activity (Chr4:52750797-52752350; not shown). Two evolutionary conserved elements are associated with *Foxi3* (*Foxi3-E1* and *-E2*; Fig. 2D), a transcription factor crucial for ear development (Birol et al., 2016; Khatri et al., 2014; Solomon et al., 2003); both drive reporter expression in two spatially and temporally distinct domains (Fig. 3E and F; see below and Fig. 4, Fig. 5). In addition, enhancers associated with the otic transcription factor *Hesx1* and with the cytokine *Cxcl14* are also active in the otic territory (Supplementary Fig. 2). In contrast to transcripts that are very strongly expressed in the otic placode, like *Spry1*, *Cxcl14* is only transiently expressed in OEPs at low levels (Supplementary Fig. S1) and this is reflected by the weak enhancer activity. Therefore, H3K27ac enrichment in response to FGF signalling identifies ear-specific enhancer regions.

### 2.4 *Foxi3* expression is controlled by two distinct regulatory regions

Foxi3 is required for otic placode formation in anamniotes and amniotes (Birol et al., 2016; Hans et al., 2007; Khatri et al., 2014; Solomon et al., 2003). While it is initially expressed in the posterior pre-placodal regions and in OEPs, it is rapidly downregulated as the otic placode forms and becomes confined to the non-otic ectoderm including the trigeminal territory and the pharyngeal arch ectoderm(Khatri and Groves, 2013). The two *Foxi3* associated enhancers seem to reflect its dynamic expression pattern and we therefore investigated their activity over time. HH4 chick embryos were co-electroporated with ubiquitously active RFP and *Foxi3-E1* driving eGFP and fluorescence was monitored at different stages. While RFP is expressed widespread, *Foxi3-E1* is begins to be activated in the posterior pre-placodal region around the 1ss and remains active in OEPs (Fig. 4A–D, c’, d’) but is shut down after the 5–6ss. Like *Foxi3* expression, enhancer activity is confined to the ectoderm (Fig. 4 c’,d’). To verify that *Foxi3-E1* is indeed inactive in the otic placode we electroporated embryos at the 5–6 somite stage and analysed them at HH12 and HH14; while RFP is expressed in the otic placode eGFP is not detected. This finding confirms that around the time of placode formation *Foxi3-E1* seems to be inactivated reflecting the change of *Foxi3* mRNA expression.

In contrast, *Foxi3-E2* becomes active slightly later. When HH5/6 embryos are co-electroporated with ubiquitously active RFP and *Foxi3-E2*-eGFP, eGFP is first observed around the 7-somite stage (HH9; Fig. 5 A, B). It remains active in the non-otic ectoderm until at least HH15 (Fig. 5 C-E, e’-e’’’’). Sections confirm that like *Foxi3* transcripts (Khatri and Groves, 2013) enhancer driven eGFP is observed in the pharyngeal arch ectoderm ventral to the otic placode or vesicle (Fig. 5 E, e’-e’’’’). Thus, both enhancers recapitulate the dynamic expression of *Foxi3* in the head ectoderm.

**Fig. 6:**
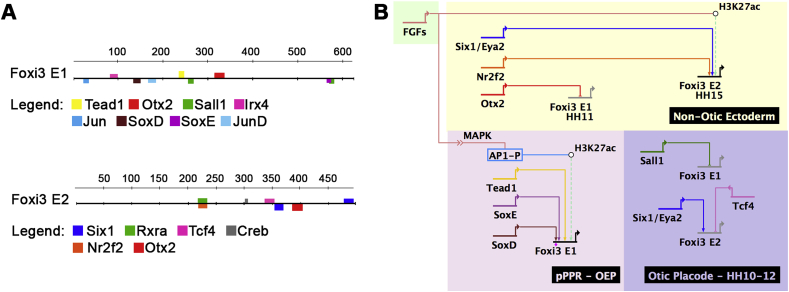
**A model for *Foxi3* regulation in the otic and non-otic ectoderm.** (A) TFBS analysis of *Foxi3-E1* and *-E2* was carried out using customized library containing motifs for otic and anterior placodal transcription factors. Putative TFBSs are coloured on the enhancer sequence at the appropriate location. (B) Putative transcriptional inputs are shown in a BioTapestry model. In the pPPR and OEP, FGF through AP1 leads to increase of H3K27ac surrounding *Foxi3-E1*, and SoxD is necessary for E1 activity (diamond indicates validated interaction). SoxE and Tead1 may be additional inputs, however, are not essential since their loss does not abolish enhancer activity. Later in the otic placode, the transcriptional repressor Sall1 mayb act as repressor to switch off *Foxi3-E1*, while anteriorly Otx2 is the predicted repressor. Furthermore, FGF promotes gain of H3K27ac around *Foxi3-E2*; Six1 and Nr2f2 are predicted inputs in the non-otic ectoderm. In the otic placode, *Foxi3-E2* is not active and may be repressed by Tcf4.

To identify putative upstream regulators of *Foxi3-E1* we examined the enrichment of transcription factor binding sites using RSAT and Clover. We identified several of motifs that correspond to factors expressed in OEPs and the otic placode (Figs. 4G and 6) including the FGF mediator cJun, SoxD and SoxE group factors, Irx4, Tead1, the late otic gene Sall1 and Otx2, which is expressed in the anterior placode territory. To identify binding sites that are required for *Foxi3-E1* activity, we deleted motifs for SoxD and SoxE alone or in combination, and for Tead1. Using a PCR based assay (Chen and Streit, 2015) we find that deletion of the SoxD motif abolishes enhancer activity completely, while SoxE or Tead1 removal show no change (Fig. 4H). Next, we electroporated ubiquitous RFP together with Foxi3-E1-ΔSoxD driving eGFP into HH6 chick embryos and assessed enhancer activity at OEP stages. We find that indeed enhancer activity is lost in the absence of the SoxD binding site (n = 7), when compared to controls (Fig. 4B–D). These findings suggest that SoxD group family members may be critical regulators of *Foxi3* expression; so far *Sox13* is the only SoxD group factor detected in otic progenitors (Chen et al., 2017). Motif enrichment analysis of *Foxi3-E2* reveals the presence of different transcription factor binding sites including for Six1, Nr2f2 and Otx2 (Fig. 6 A), which are expressed in non-otic ectoderm including the trigeminal and epibranchial placodes.

Together this analysis allows us to propose a model for temporal and spatial *Foxi3* regulation (Fig. 6). We propose that in the posterior pre-placodal region, *Foxi3* is activated by FGF signalling via AP1 (see below), which increases H3K27ac around *Foxi3-E1*. The SoxD group family member Sox13 is also directly upregulated by FGF signalling (Fig. 1) and the requirement of the SoxD motif for enhancer activity suggest that Sox13 participates in its activation, while Tead1 and SoxE group members may be involved but are not essential. At placode stages, when *Foxi3* is downregulated in the otic territory, the transcriptional repressor *Sall1* becomes expressed and may contribute to *Foxi3-E1* shut-down, while Otx2 may help repress it anteriorly. *Foxi3-E2* is never active in the otic placode and the transcriptional repressor Tcf4 may prevent its activation. In the non-otic ectoderm *Foxi3-E2* is also activated by FGF signalling and deposition of H3K27ac and may receive input from the Six1/Eya2 complex and Nr2f2.

### 2.5 Unique transcription factor motifs define OEP enhancers

**Fig. 7:**
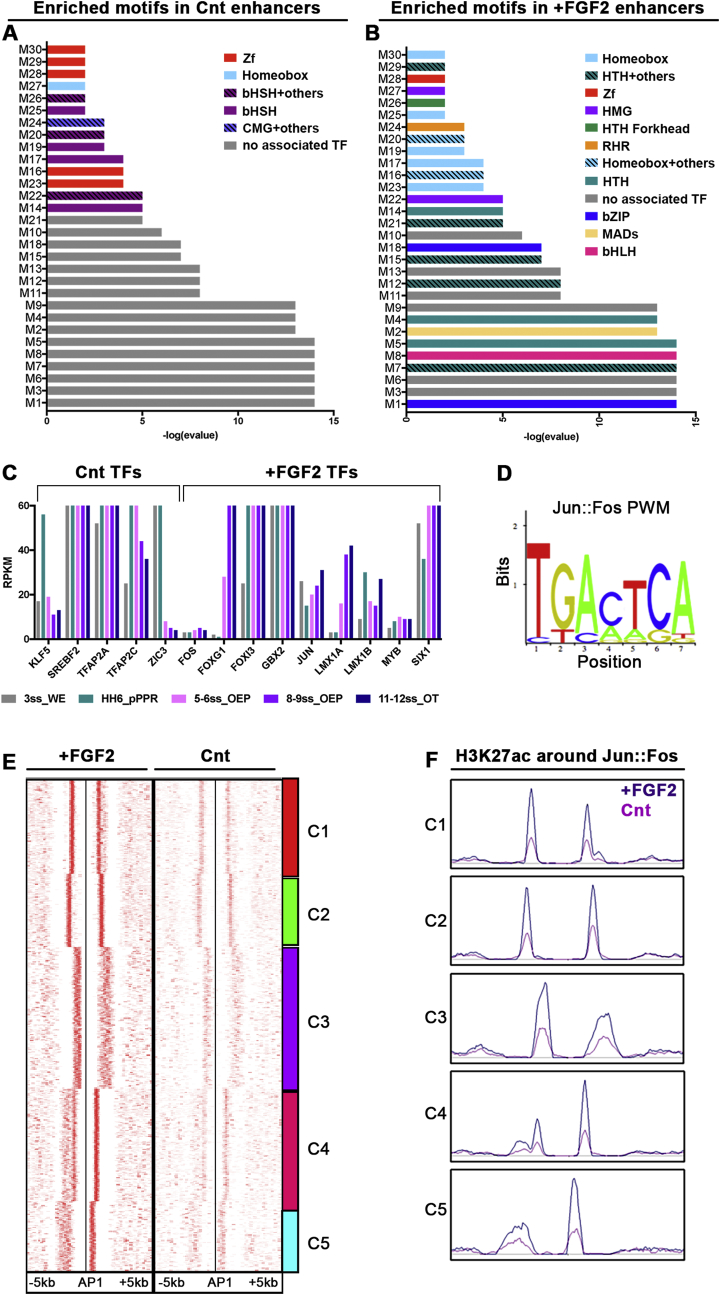
**Distinct transcription factor binding sites characterise sensory progenitor and OEP enhancers.** (A–B) Motif enrichment analysis using RSAT of enhancers active in control (Cnt; A) and FGF2 (B) treated pre-placodal cells. Transcription factor families corresponding to the enriched motifs (M1-M30) are colour coded, -log (evalue) is plotted on the y axis. (C) Data from Chen and colleagues (Chen et al., 2017) show the expression levels (RPKM) of transcription factors corresponding to enriched motifs in the 3ss whole embryo (grey), in 0ss posterior pre-placodal cells (green), 5–6ss OEPs (pink), 8–9ss OEPs (violet) and the 11–12ss otic placode (blue). (D) Jun:Fos (Ap1) Position Weight Matrix (PWM) logo; note: the Ap1 motif is highly enriched in FGF2-treated samples. (E) Distribution of H3K27ac peaks surrounding AP1 motifs in FGF2-treated and control pre-placodal cells; SeqMINER view of the heatmaps. (F) H3K27ac peak profiles around the Ap1 motif in FGF2 treated (violet) and control (pink) pre-placodal cells in a window of −/+5 ​kb.

Enhancers serve as transcription factor hubs that integrate different transcriptional inputs to activate cell fate specific gene expression. To investigate whether a unique transcriptional signature is associated with OEP genes, we performed motif enrichment analysis. Using RSAT matrix-scan we scanned for TF binding sites enriched in enhancers active in FGF2-treated versus control cells and vice versa using known motifs from JASPAR and TRANSFAC libraries. Enhancers uniquely active in controls cells lose H3K27ac upon FGF treatment and are preferentially associated with FGF-repressed genes (see above). These show enrichment of TFAP2 (Supplementary File 2 - Cnt_M14_M17_M26) and unknown motifs (Fig. 7A; Supplementary File 2), as well as Zic1/3, Srebf2 and Klf5 binding sites. Based on published transcriptome data (Chen et al., 2017) the corresponding transcription factors *TFAP2a, -b, -c* and *-e*, *Klf5* and *Zic1/3* are expressed in pre-placodal cells, while *Srebf2* seems to be expressed ubiquitously (Fig. 7C; Supplementary Fig. 6). Together, these factors may be important for the regulation of sensory progenitor genes or the activation of genes required for alternative fates. Indeed, TFAP family members control both neural crest and sensory progenitor fates at the neural plate border (Hoffman et al., 2007; Knight et al., 2003; Li and Cornell, 2007; Qiao et al., 2012), while Zic transcription factors together with Pax3 discriminate between both fates (Hong and Saint-Jeannet, 2007).

In contrast, enhancers associated to OEP genes show a different motif signature. Motifs corresponding to homeobox and winged-helix/forkhead domain transcription factors are highly enriched (Fig. 7B; Supplementary File 3 FGF2_M5_12_15_21_16_20_29), as are binding sites for basic-loop-helix factors (Fig. 4B; Supplementary File 3 FGF2_M8), for the transcription factor Myb (Fig. 7B; Supplementary File 3 FGF2_M7) and the FGF signalling mediators of the Jun and Fos families (Fig. 4B; Supplementary File 3 FGF2_M18). Many of the corresponding transcription factors are expressed in OEPs and required for their specification including *Dlx5/6/3*, *Gbx2*, *Lmx1a*, *Six1* and *Foxi3* (Birol et al., 2016; Brugmann et al., 2004; Chen et al., 2017; Christophorou et al., 2009; Kaji and Artinger, 2004; Khatri et al., 2014; Luo et al., 2001; McLarren et al., 2003; Sato et al., 2010), while others play a role at later stages of ear development (e.g. *Foxg1*) (Hwang et al., 2009) and yet others are expressed in OEPs, but their function has not been investigated (*FoxP1*, *FoxC2*, *FoxK2*; Chen et al., 2017). Thus, a unique signature of transcription factor binding sites defines OEP enhancers. The corresponding transcription factors are known to control otic identity, while others represent new putative upstream regulators.

### 2.6 AP1 recruits the histone acetyl-transferase p300 to ear-specific enhancers

The Fos/Jun transcription factors are mediators of FGF signalling and form homo- or heterodimers (AP1) to activate downstream targets. The AP1 motif is differentially associated with FGF2-induced elements (Fig. 7B; Supplementary File 3 – FGF2_M18) and is indeed present near the enhancers tested *in vivo* (Fig. 3A, D; Supplementary Fig. 2A). Examining the distribution of H3K27Ac within 5 kb surrounding AP1 motifs reveals a strong correlation in FGF-treated cells, while the same regions are depleted in controls (Fig. 7E and F). Therefore, enhancers activated in response to FGF show significant enrichment of AP1 binding sites as compared to controls. Clustering and peak density plots identifies five different clusters (C1–C5) that differ in the location of H3K27ac marks with respect to AP1 motifs (Fig. 7E and F). Functional annotation of these clusters shows enrichment of terms like ear morphogenesis, inner ear development, sense organ development and, importantly, MAPK signalling (Supplementary Fig. 7). This finding suggests a major difference in enhancer activation during OEP induction can be attributed to the FGF pathway with AP1 as a mediator.

**Fig. 8:**
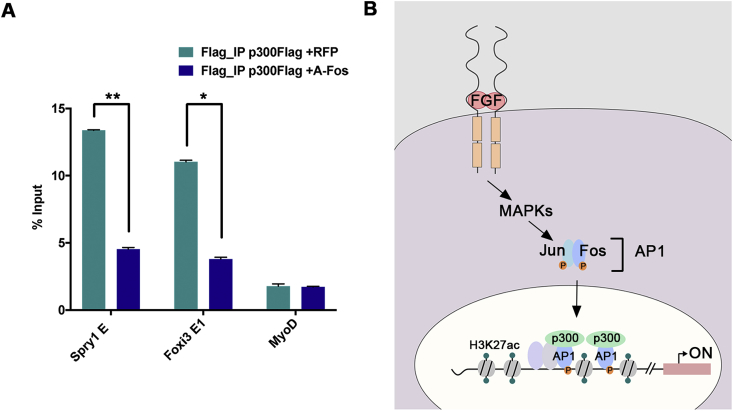
**Ap1 recruits p300 to Spry1 and Foxi3 enhancers.** (A) p300 occupies OEP *Spry1-E* and *Foxi3-E1*. Flag-tagged p300 together with a plasmid containing RFP (controls) or dominant negative A-Fos (experimental) was electroporated into future OEPs at HH6. After 6 h targeted otic placodes were dissected and ChIP was performed with anti-Flag antibodies. p300 occupies *Spry1* and *Foxi3* enhancers active in OEPs, but does not bind to the muscle gene *MyoD*. In the presence of A-Fos, p300 binding is abolished on both OEP enhancers. (B) A model for OEP gene activation in response to FGF signalling. See text for details.

What is the mechanism by which FGF and AP1 activate OEP enhancer regions? Acetylation of H3K27 is mediated by histone acetyl-transferases (HAT) like p300. To assess whether p300 occupies otic enhancers, we selected two experimentally verified enhancers, *Spry1-E* and *Foxi3-E1*, with a significant gain of H3K27ac in response to FGF activation. We electroporated p300-Flag into ear progenitors and performed anti-Flag ChIP-qPCR from targeted tissue. Indeed, p300 is bound to both enhancers, but is absent from the muscle gene *MyoD* (Fig. 8A).

AP1 is known to interact with p300 (Crish and Eckert, 2008), raising the possibility that it recruits p300 to OEP enhancers to facilitate histone acetylation. To test this, we made use of a dominant negative form of Fos (acidic-Fos: a-Fos; Biddie et al., 2011), a truncated version of Fos that maintains its interaction with Jun but cannot bind DNA. HH6 chick embryos were electroporated with p300-Flag together with a-Fos or RFP (control) constructs; targeted otic progenitors were dissected and processed for ChIP-qPCR using anti-flag antibodies. While p300 binding to *Spry1-E* and *Foxi3-E1* is observed in controls, this is inhibited in the presence of a-Fos (Fig. 8A). No binding is observed at the MyoD enhancer, a transcript not expressed in ear progenitors. These findings suggest the model that in response to FGF signalling, AP1 recruits the HAT p300, which in turn increases acetylation of H3K27 at ear-specific enhancers and as a result rapidly induces their expression (Fig. 8B).

## 3 Discussion

### 3.1 FGF signalling mediates enhancer activation in otic-epibranchial progenitors

In the embryo, cell fate choices are mediated by temporally and spatially controlled signals that activate distinct transcriptional programmes in responding cells. The key elements that integrate this information are non-coding, cis-regulatory regions that control cell type specific gene expression, which in turn implement fate decisions (Banerji et al., 1981; Beagrie and Pombo, 2016; Pennacchio et al., 2013). Enhancer elements are characterised by specific epigenetic signatures, however how transient signalling cues during *in vivo* development affect the chromatin landscape is not well understood. Here we study the changes in H3K27ac, a histone mark associated with active enhancers (Creyghton et al., 2010; Kharchenko et al., 2011; Rada-Iglesias et al., 2011, 2012; Zentner et al., 2011), as pre-placodal cells acquire OEP fate in response to FGF signalling. We find that FGF rapidly induces dynamic changes at thousands of genomic regions with a marked gain of H3K27 acetylation near OEP genes. We show that these act as enhancer elements that drive OEP-specific gene expression *in vivo* and enhancer associated transcripts are indeed rapidly upregulated by FGF. In contrast, genomic regions surrounding genes normally absent in OEPs lose H3K27 acetylation, and these transcripts are repressed by FGF. Thus, we have identified thousands of FGF-activated and -repressed regulatory elements during OEP induction providing a rich resource to explore the gene regulatory network that controls ear development.

### 3.2 AP1 mediates p300 recruitment to FGF activated enhancers

FGF signalling activates gene expression in responding cells within minutes, and our studies provide a molecular mechanism underlying this response. In ear progenitors, FGF is mediated by the MAP-kinase pathway (Yang et al., 2013a) leading to phosphorylation of the transcription factors Jun and Fos, which work as a heterodimer AP1 to activate gene expression (Ornitz and Itoh, 2015; Tsang and Dawid, 2004; Yang et al., 2003, 2013b). The AP1 binding motif is strongly enriched in FGF-activated enhancers, and we show that AP1 is required to recruit the histone acetyltransferase p300 to at least some ear enhancers. Indeed, AP1 and p300 physically interact (Crish and Eckert, 2008), and inhibition of p300 prevents induction of some FGF response genes in pre-placodal cells. This supports the idea that p300 activity and subsequent increased histone acetylation is required to activate OEP genes. A similar mechanism has recently been reported in HeLa cells (O’Donnell et al., 2008). Here, in response to MAP kinase signalling, Elk is phosphorylated and together with p300 changes the histone acetylation state, which in turn allows the recruitment of other transcription factors and subsequent gene activation. It is therefore possible that increased acetylation in OEPs enhances chromatin accessibility allowing other otic transcription factors to access target enhancers, where they may cooperate with AP1 to activate target transcription. Motif enrichment analysis of FGF-induced ear enhancers suggests that homeobox and winged-helix/forkhead proteins are possible cooperators. Of those, the homebox genes *Gbx2*, *Six1* and *Lmx1a* and the forkhead gene *Foxi3* are indeed expressed in OEPs and are required for their specification and/or vesicle formation (Birol et al., 2016; Brugmann et al., 2004; Chen et al., 2017; Christophorou et al., 2009; Kaji and Artinger, 2004; Khatri et al., 2014; Luo et al., 2001; McLarren et al., 2003; Sato et al., 2010). In addition, we have recently shown that Myb recruits the histone demethylase Lsd1 to OEP gene promoters to maintain their expression after initial specification (Ahmed and Streit, 2018), while our current results suggest that Myb may also cooperate with AP1 at OEP enhancer regions.

Furthermore, AP1 is known to influence chromatin accessibility using different mechanisms and may act as a pioneer factor to mediate chromatin opening. In mouse embryonic fibroblasts, AP1 can bind to nucleosome-occupied regulatory elements together with cell type specific transcription factors (Vierbuchen et al., 2017). Together, they recruit the SWI/SNF complex BAF, which repositions nucleosomes to increase enhancer accessibility. Likewise, AP1 is required for glucocorticoid induced transcription by priming glucocorticoid receptor target sites and maintaining their accessibility before the receptor can be recruited (Biddie et al., 2011). Whether similar mechanisms also operate in OEPs in response to FGF signalling remains to be uncovered.

FGF signalling not only promotes OEP fate, but also shuts down alternative differentiation programmes. For example, we have previously shown that the lens programme is inhibited in OEPs by FGFs with the lens marker *Pax6* being a direct target (Anwar et al., 2017; Bailey et al., 2006). Here we observe a significant decrease of H3K27ac in the Pax6 locus and a complementary increase of H3K27me3. Recent evidence suggests that AP1 may mediate transcriptional repression by recruiting histone deacetylases (Miotto et al., 2006; Mittelstadt and Patel, 2012), which subsequently allows H3K27 methylation. Interestingly, in the neural tube FGF signalling promotes compaction of the Pax6 locus mediated by polycomb repressive complexes (Patel et al., 2013; Semprich et al., 2019), while inhibition of Erk1/2 results in dissociation of this complex from the Pax6 TSS. It is therefore possible that in OEPs increased H3K27me3 deposition at the Pax6 locus allows recruitment of the polycomb repressor complex to repress *Pax6*, a key gene for lens formation. Thus, AP1 function depends on a fine balance between activator and repressor complexes, presumably controlled by the availability of different enhancer specific co-factors.

### 3.3 OEP induction: new transcription factors downstream of FGF signalling

It is well established that FGF signalling activates the inner ear programme by inducing otic-epibranchial progenitors from a pool of multipotent precursors. While continued FGF activation promotes epibranchial identity, Notch together with Wnt signalling establishes the otic placode (Freter et al., 2008; Ohyama et al., 2006; for review: Chen and Streit, 2013; Groves and Fekete, 2012; Ladher, 2017; Ohyama et al., 2007; Whitfield, 2015; Whitfield et al., 2002). Here we identify new FGF-response genes and their associated enhancers. Using network inference approaches we have recently proposed that FGFs initially enhances the transcription of genes already expressed in pre-placodal cells, which then act in a positive feedback loop to increase their own expression (Anwar et al., 2017). Downstream of this circuit, *Pax2* and other transcription factors are activated to regulate OEP identity while repressing alternative fates. Here, we show that FGF leads to the activation of more than 20 transcription factors, many of which are expressed in OEPs, but absent from other placodes. These include the known targets *Etv4* and *-5* and the FGF antagonists *Spry-1* and *-2*, as well as many new FGF-responsive factors like the direct targets *Sox13*, *Tead3* and *Klf7*. Transcription factor binding motifs for these factors are overrepresented in ear enhancers suggesting that they may control the ear identity. Our finding that SoxD motifs are required for the activity of the *Foxi3-E1* enhancers in OEPs, supports the idea that SoxD factors like Sox13 play an important role in early otic specification. Furthermore, in *Xenopus* and mouse, the transcription factor *Klf7* is expressed in the otic placode (Gao et al., 2015; Laub et al., 2001). Klf7 has been implicated in stem cell renewal, as well as cell cycle exit of neuronal precursors (Jeon et al., 2016; Kajimura et al., 2007; Laub et al., 2005, 2006), however its function in ear development remains unknown.

In summary, during the induction of otic-epibranchial progenitors, FGF signalling rapidly activates downstream target genes by increasing H3K27 acetylation at enhancer regions. FGF activity is mediated by the Jun/Fos heterodimer AP1, which recruits the acetyltransferase p300 to OEP enhancers. The genome-wide identification of FGF-responsive OEP enhancer regions forms the basis to reconstruct the otic induction network and may shed light on other FGF-mediated processes in the ear.

## 4 Materials and methods

### 4.1 Embryo manipulation and explant culture

Experiments on chick embryos prior to E10 do not require a home office license or institutional approval and were carried out according to the institutional guidelines. Fertilized chicken eggs were obtained from Winter Farm (Herts, UK) and incubated in a humidified incubator at 38 °C until reaching the desired stage (Hamburger and Hamilton, 1951). For electroporation, embryos were cultured using the filter culture method (Chapman et al., 2001). To isolate sensory progenitor explants, embryos were collected in Tyrode’s saline, mesoderm and endoderm layers were removed, pre-placodal cells were dissected using fine needles, and cultured in DMEM/N2 supplemented with FGF2 (0.25 ng/μl; R&D), DMSO or cycloheximide for 3 or 6 h.

### 4.2 Enhancer cloning and electroporation

Putative enhancer elements were amplified from chick genomic DNA and inserted into pTK vector containing eGFP reporter and a TK minimal promoter (Kondoh and Uchikawa, 2008). Cloning primers are listed in Supplementary Table 1. Flagged tagged p300 vector was kindly provided by Michael O. Hottiger (Hasan et al., 2001). For electroporation, embryos on filter paper were transferred into an electroporation chamber containing a 2 × 2mm platinum electrode. DNA (3 μg/μl reporter DNA pTK eGPF and 1.5 μg/μl pCAB RFP, 0.1% fast green; 1 μg/μl p300-Flag and 1.5 μg/μl pCAB RFP, 0.1% fast green) was injected by air pressure between the vitelline membrane and embryonic ectoderm, a platinum electrode (2 × 1 mm) was placed above the target area and 5 pulses of 4 V for 50 ms with 750 ms intervals were applied using Intracel TSS20 OVODYNE pulse generator. Embryos were cultured overnight and imaged using an inverted fluorescent microscope (Zeiss Axiovert). Selected embryos were cryo-sectioned (8 μm) and processed for immunohistochemistry with anti-mCherry (1:200, rabbit, ab167453, Abcam) and anti-GFP (1:200, mouse, A11120, Invitrogen/Life Tech), followed by secondary antibody Alexa Fluor® 488 (1:1000, A11001, Invitrogen/Life Tech) and Alexa Fluor® 568 (1:1000, A11036, Invitrogen/Life Tech). Sections were imaged with Leica SP5 confocal microscope.

### 4.3 Enhancer mutagenesis and one-step RT-PCR

For *Foxi3* enhancers, transcription factor binding site analysis was carried out using RSAT matrix-scan (Turatsinze et al., 2008) and Clover (Frith et al., 2004), with JASPAR (Sandelin et al., 2004) and TRANSFAC (Matys et al., 2006) customized library containing enriched PPR, otic, lens and trigeminal transcription factors. As control, sequence shuffling was carried out 1000 times and subsequently p-values were calculated to determine significant binding sites. For RSAT matrix-scan, the default p-value of 1e^−4^ and for Clover, a p-value of 0.01 was used. Predicted binding sites relevant corresponding to factors expressed in the otic cells were considered for downstream analysis.

Site-directed mutagenesis of *Foxi3-E1* ΔSoxD, *Foxi3-E1* ΔTead1 and *Foxi3-E1* ΔSoxE/D was obtained by PCR-driven overlap extension (Heckman and Pease, 2007). In brief, mutagenic primers (P2 and P3) and flanking primers (P1 and P4) were used to generate intermediate PCR products P1–P2 and P3–P4, that are overlapping fragments of the entire product P1–P4. Products P1–P2 and P3–P4 when denatured are used as DNA template for the second PCR; strands of each product hybridize at their overlapping, complementary regions that contain the desired deletion. Amplification of product P1–P4 in the second PCR is driven by primers P1 and P4. Final product P1–P4 was inserted into tagged version of pTK vector containing eGFP reporter (Chen and Streit, 2015) by standard T/A cloning. *Foxi3-E1* ΔSoxE deletion was obtained by PCR amplification of *Foxi3-E1* with the exclusion of the most terminal part of the enhancer (SoxE binding site), using the primers listed in Supplementary Table 2, the PCR product was inserted in pTK vector containing eGFP reporter Tag3 (Chen and Streit, 2015). Mutagenesis was verified by Sanger sequencing.

For RT-PCR screening, the four mutagenized enhancers and *Foxi3-E1* (Tag6) were mixed each at a final concentration of 0.3 ​μg/μl. The plasmid pool was electroporated together with pCAB RFP as control plasmid. Two RFP ​+ ​OEP tissues were dissected and processed for RT-PCR with unique barcode and common reverse primers, as previously described (Chen and Streit, 2015).

### 4.4 NanoString nCounter analysis

For NanoString nCounter analysis, eight to ten explants per condition were lysed in 5 μl lysis buffer (Ambion) and analysed by nCounter® Analysis System (Life Sciences) using a customized probe set of 216 genes (Chen et al., 2017). Total RNA was hybridized with capture and reporter probes at 65 °C overnight. Target/probe complexes were washed and immobilized according to the nCounter Gene Expression Assay Manual, and data were collected by the nCounter Digital Analyzer. Experiments were repeated on three independent occasions and data were analysed following company instructions. A cut-off of fold change ≥ 1.25 and ≤ 0.75 was used to identify upregulated and downregulated genes, respectively, in combination with a p-value ≤ 0.05 (unpaired *t*-test).

### 4.5 *In situ* hybridization

The following chick plasmids were used to generate Digoxigenin–labeled antisense probes: Etv4 (a kind gift from M. Bronner); Spry1 obtained from Life Technologies; Klf7 ChEST376015; Sox13 ChEST437d11; Pax2 (a kind gift from M. Golding) Pax6 (a kind gift from A. Bang) and Cxcl14 ChEST896P24. RNA probes were synthesized with T7, T3 or SP6 RNA polymerase (Roche). Whole mount or explant *in situ* hybridization was performed as described previously (Streit and Stern, 2001).

### 4.6 Chromatin immunoprecipitation (ChIP)

Around 100 posterior pre-placodal cells from HH6 were cultured for 6 h in the absence or presence of FGF2. Explants were dissociated in 0.5 ml of Nuclei Extraction Buffer (NEB; 0.5% NP-40, 0.25% Triton X-100, 10 mM Tris-HCl pH 7.5, 3 mM CaCl_2_, 0.25 M Sucrose, 1 mM DTT, 0.2 mM PMSF, 1X Protease Inhibitor) by homogenisation and crosslinked using 1% formaldehyde for 9 min at room temperature. 0.125 M glycine final concentration was used to stop fixation. Samples were pelleted and stored at −80 °C. 100 μl of Dynal magnetic beads (Protein A, Novex Life Technologies) were incubated with 1 ml of blocking buffer (1X PBS with 0.5% BSA) for 2 min, resuspended in 250 μl of blocking buffer and respective antibodies were added (1 μg/μl Rabbit anti-IgG Millipore – CA92590; 1 μg/μl Rabbit anti-H3K27ac Abcam – ab4729; 2.5 μg Rabbit anti-H3K27me3, Cell signalling – C36B11; 3 μg anti-Flag, Sigma – F3165). Antibody and beads were incubated overnight rotating at 4 °C, and excess of antibody was removed by washes in blocking buffer. Crossed-linked pellets were defrosted, washed in Nuclei Extraction Buffer and nuclei were extracted by homogenisation. Samples were lysed in 1% SDS, 50 mM Tris-HCl (pH 8), 10 mM EDTA supplemented with 20 μl Protease Inhibitor (7x PI; Roche) for 1 h. Prior to sonication 280 μl of ChIP dilution buffer (0.01% SDS; 16.7 mM Tris-HCl pH 8; 1.2 mM EDTA; 167 mM NaCl; supplemented with PMSF, DTT and PI) was added, samples were sonicated to generate fragments of 200–600bp using 12 cycles of 15sec on and 30sec off at 40% amplitude (SONICS, Vibra Cell™). The resulting chromatin was diluted 4x and divided across different samples, 1/10th of sonicated chromatin was kept frozen as input control. Beads were washed 9 times in RIPA buffer containing 50 mM Hepes-KOH (pH 8), 500 mM LiCl, 1 mM EDTA, 1% NP-40, 1.7% Na-deoxycholate supplemented with PI. A final wash was performed using 1x TE, 50 mM NaCl and the chromatin was eluted in 50 mM Tris-HCl (pH 8), 10 mM EDTA and 1% SDS. Reverse-crosslinking was carried out at 65 °C overnight. Chromatin was diluted using 1xTE and incubated at 37 °C for 1 h with RNAse A 0.4 mg/μl, followed by proteinase K digestion for 1 h at 55 °C. Chromatin was purified using phenol: chloroform: isoamyl alcohol and resuspended in 60 μl of water. ChIPed chromatin was processed for next generation sequencing or analysed by qPCR. For qPCR Ct values for each precipitation were extracted and normalized to input chromatin, primer sequences are listed in Supplementary Table 3. For next generation sequencing chromatin was amplified with a step of linear amplification following the protocol described in (Adli and Bernstein, 2011). Library preparation was performed at the UCL Genomics, Institute of Child Health following the protocol used for nano-ChIP-seq (Adli and Bernstein, 2011) with the exception that only 12 cycles were used in the PCR amplification. Data have been deposited at Gene Expression Omnibus (accession number: GSE137664).

### 4.7 ChIP-seq data analysis

Sequence quality was assessed using FastQC. During each ChIP-seq experiment, an amplification step was carried out that is reported to produce mismatches at the first 9bp due to random priming (Adli and Bernstein, 2011). Therefore, as the sequence quality was poor these 9bp were trimmed. Further trimming was performed to improve alignment if sequence quality from the 3′ end was poor. Reads were aligned to the chick genome Galgal4.71 using Novoalign (Novocraft 2.08.01, http://www.novocraft.com/products/novoalign/) and uniquely aligned sequences were used for peak calling using Homer (Heinz et al., 2010) and MACS2 (Zhang et al., 2008). For Homer, a fold change of 1.5 relative to input and a False Discovery Rate (FDR) of 0.01 were used. For MACS2, a FDR of 0.05 as suggested in the MACS manual to obtain broad peaks (characteristic of histone peaks) and a default p-value of 1e-5 were used. Genome wide analysis was performed on Homer called peaks. Following peak-calling, putative enhancers were identified in the following way: regions of up to 3 ​kb flanked by H3K27ac and devoid of H3K27me3 peaks were identified and assigned to the nearest gene using gene annotations from Ensembl (Galgal4.71) and refGene (Nov. 2011 ICGSC Gallus_gallus-4.0/galGal4). Read distributions around transcription start site (TSS) or the centre of a putative enhancer was plotted using ’annotatePeaks’. Putative enhancers for ​+ ​FGF2-treated and control samples were compared to find common and unique putative enhancers using the R package ChIPpeakAnno (Zhu et al., 2010). Putative enhancers in +FGF2 and Cnt were considered to be overlapping and common if they had a 0 bp gap between them, otherwise they were considered to be unique to the respective condition. Genes associated with unique ​+ ​FGF2 and Cnt putative enhancers were subjected to Gene Ontology (GO) analysis using DAVID (DAVID Bioinformatics Resources 6.7) (Huang et al., 2009a, b). All ChIP-seq data were viewed in the IGB browser (Nicol et al., 2009), and deposited in Gene Expression Omnibus (accession number: TBC).

### 4.8 Transcription factor binding site analysis

Transcription factor binding site analysis was carried out using RSAT peak-motifs (Thomas-Chollier et al., 2012a, 2012b) and JASPAR (Sandelin et al., 2004) and TRANSFAC (Matys et al., 2006) libraries. To obtain significant binding sites, an e-value ≤ 0.05 was considered.

### 4.9 Conservation

Multiple alignments between 21 amniotes were obtained from Ensemble PECAN (Paten et al., 2008). Additionally, DREiVe (Khan et al., 2012), a motif-discovery algorithm was used to identify putative regulatory regions 300 KB upstream and downstream of selected FGF-response genes. It reports regulatory regions as clusters of short conserved motifs of 8 bp in a 300 bp window. DREiVe does not depend on sequence alignment, is able to identify re-arrangements of motifs within regulatory elements and does not require prior information of transcription factor binding sites. Regions that were conserved in 7 out of 9 species (human, horse, cow, rabbit, mouse, opossum, platypus, chick and lizard) were considered as putative enhancers. These tracks were then loaded into IGB browser to view with Chip-seq data.

## Competing interests

The authors declare no competing or financial interests.

## Author contributions

A.S. obtained the funding and managed the project; A.S. designed the experiments together with M.T.; M.T. conducted most experiments and analysed the data together with A.S.; M.A. performed all bioinformatics analysis; M. Ahmed performed ChIP-qPCR in Fig. 5 and performed the Foxi3-E1ΔSoxD analysis together with M.T. M.T. and M. A. prepared figures. A.S. wrote the manuscript together with M. T.

## References

[bib1] Abello G., Khatri S., Radosevic M., Scotting P.J., Giraldez F., Alsina B. (2010). Independent regulation of Sox3 and Lmx1b by FGF and BMP signaling influences the neurogenic and non-neurogenic domains in the chick otic placode. Dev. Biol..

[bib2] Adamska M., Herbrand H., Adamski M., Kruger M., Braun T., Bober E. (2001). FGFs control the patterning of the inner ear but are not able to induce the full ear program. Mech. Dev..

[bib3] Adli M., Bernstein B.E. (2011). Whole-genome chromatin profiling from limited numbers of cells using nano-ChIP-seq. Nat. Protoc..

[bib4] Ahmed M., Streit A. (2018). Lsd1 interacts with cMyb to demethylate repressive histone marks and maintain inner ear progenitor identity. Development.

[bib5] Ahrens K., Schlosser G. (2005). Tissues and signals involved in the induction of placodal Six1 expression in Xenopus laevis. Dev. Biol..

[bib6] Alsina B., Abello G., Ulloa E., Henrique D., Pujades C., Giraldez F. (2004). FGF signaling is required for determination of otic neuroblasts in the chick embryo. Dev. Biol..

[bib7] Alvarez Y., Alonso M.T., Vendrell V., Zelarayan L.C., Chamero P., Theil T., Bosl M.R., Kato S., Maconochie M., Riethmacher D., Schimmang T. (2003). Requirements for FGF3 and FGF10 during inner ear formation. Development.

[bib8] Anwar M., Tambalo M., Ranganathan R., Grocott T., Streit A. (2017). A gene network regulated by FGF signalling during ear development. Sci. Rep..

[bib9] Bailey A.P., Bhattacharyya S., Bronner-Fraser M., Streit A. (2006). Lens specification is the ground state of all sensory placodes, from which FGF promotes olfactory identity. Dev. Cell.

[bib10] Banerji J., Rusconi S., Schaffner W. (1981). Expression of a beta-globin gene is enhanced by remote SV40 DNA sequences. Cell.

[bib11] Beagrie R.A., Pombo A. (2016). Gene activation by metazoan enhancers: diverse mechanisms stimulate distinct steps of transcription. Bioessays.

[bib12] Biddie S.C., John S., Sabo P.J., Thurman R.E., Johnson T.A., Schiltz R.L., Miranda T.B., Sung M.H., Trump S., Lightman S.L., Vinson C., Stamatoyannopoulos J.A., Hager G.L. (2011). Transcription factor AP1 potentiates chromatin accessibility and glucocorticoid receptor binding. Mol. Cell.

[bib13] Birol O., Ohyama T., Edlund R.K., Drakou K., Georgiades P., Groves A.K. (2016). The mouse Foxi3 transcription factor is necessary for the development of posterior placodes. Dev. Biol..

[bib14] Boyle A.P., Davis S., Shulha H.P., Meltzer P., Margulies E.H., Weng Z., Furey T.S., Crawford G.E. (2008). High-resolution mapping and characterization of open chromatin across the genome. Cell.

[bib15] Brugmann S.A., Pandur P.D., Kenyon K.L., Pignoni F., Moody S.A. (2004). Six1 promotes a placodal fate within the lateral neurogenic ectoderm by functioning as both a transcriptional activator and repressor. Development.

[bib16] Calo E., Wysocka J. (2013). Modification of enhancer chromatin: what, how, and why?. Mol. Cell.

[bib17] Canning C.A., Lee L., Luo S.X., Graham A., Jones C.M. (2008). Neural tube derived Wnt signals cooperate with FGF signaling in the formation and differentiation of the trigeminal placodes. Neural Dev..

[bib18] Catarino R.R., Stark A. (2018). Assessing sufficiency and necessity of enhancer activities for gene expression and the mechanisms of transcription activation. Genes Dev..

[bib19] Chapman S.C., Collignon J., Schoenwolf G.C., Lumsden A. (2001). Improved method for chick whole-embryo culture using a filter paper carrier. Dev. Dynam..

[bib20] Chen J., Streit A. (2013). Induction of the inner ear: stepwise specification of otic fate from multipotent progenitors. Hear. Res..

[bib21] Chen J., Streit A. (2015). A medium-scale assay for enhancer validation in amniotes. Dev. Dynam..

[bib22] Chen J., Tambalo M., Barembaum M., Ranganathan R., Simoes-Costa M., Bronner M.E., Streit A. (2017). A systems-level approach reveals new gene regulatory modules in the developing ear. Development.

[bib23] Chen Y.J., Wang Y.N., Chang W.C. (2007). ERK2-mediated C-terminal serine phosphorylation of p300 is vital to the regulation of epidermal growth factor-induced keratin 16 gene expression. J. Biol. Chem..

[bib24] Christophorou N.A., Bailey A.P., Hanson S., Streit A. (2009). Activation of Six1 target genes is required for sensory placode formation. Dev. Biol..

[bib25] Creyghton M.P., Cheng A.W., Welstead G.G., Kooistra T., Carey B.W., Steine E.J., Hanna J., Lodato M.A., Frampton G.M., Sharp P.A., Boyer L.A., Young R.A., Jaenisch R. (2010). Histone H3K27ac separates active from poised enhancers and predicts developmental state. Proc. Natl. Acad. Sci. U. S. A..

[bib26] Crish J.F., Eckert R.L. (2008). Synergistic activation of human involucrin gene expression by Fra-1 and p300--evidence for the presence of a multiprotein complex. J. Investig. Dermatol..

[bib27] Doetzlhofer A., Basch M.L., Ohyama T., Gessler M., Groves A.K., Segil N. (2009). Hey2 regulation by FGF provides a Notch-independent mechanism for maintaining pillar cell fate in the organ of Corti. Dev. Cell.

[bib28] Freter S., Muta Y., Mak S.S., Rinkwitz S., Ladher R.K. (2008). Progressive restriction of otic fate: the role of FGF and Wnt in resolving inner ear potential. Development.

[bib29] Frith M.C., Fu Y., Yu L., Chen J.F., Hansen U., Weng Z. (2004). Detection of functional DNA motifs via statistical over-representation. Nucleic Acids Res..

[bib30] Gao Y., Cao Q., Lu L., Zhang X., Zhang Z., Dong X., Jia W., Cao Y. (2015). Kruppel-like factor family genes are expressed during Xenopus embryogenesis and involved in germ layer formation and body axis patterning. Dev. Dynam..

[bib31] Groves A.K., Fekete D.M. (2012). Shaping sound in space: the regulation of inner ear patterning. Development.

[bib32] Gruda M.C., Kovary K., Metz R., Bravo R. (1994). Regulation of Fra-1 and Fra-2 phosphorylation differs during the cell cycle of fibroblasts and phosphorylation in vitro by MAP kinase affects DNA binding activity. Oncogene.

[bib33] Hamburger V., Hamilton H.L. (1951). A series of normal stages in the development of the chick embryo. J. Morphol..

[bib34] Hammond K.L., Whitfield T.T. (2011). Fgf and Hh signalling act on a symmetrical pre-pattern to specify anterior and posterior identity in the zebrafish otic placode and vesicle. Development.

[bib35] Hans S., Christison J., Liu D., Westerfield M. (2007). Fgf-dependent otic induction requires competence provided by Foxi1 and Dlx3b. BMC Dev. Biol..

[bib36] Haque K., Pandey A.K., Zheng H.W., Riazuddin S., Sha S.H., Puligilla C. (2016). MEKK4 signaling regulates sensory cell development and function in the mouse inner ear. J. Neurosci..

[bib37] Hasan S., Hassa P.O., Imhof R., Hottiger M.O. (2001). Transcription coactivator p300 binds PCNA and may have a role in DNA repair synthesis. Nature.

[bib38] Heckman K.L., Pease L.R. (2007). Gene splicing and mutagenesis by PCR-driven overlap extension. Nat. Protoc..

[bib39] Heinz S., Benner C., Spann N., Bertolino E., Lin Y.C., Laslo P., Cheng J.X., Murre C., Singh H., Glass C.K. (2010). Simple combinations of lineage-determining transcription factors prime cis-regulatory elements required for macrophage and B cell identities. Mol. Cell.

[bib40] Hoffman T.L., Javier Anna L., Campeau Shelley A., Knight Robert D., Schilling Thomas F. (2007). Tfap2 transcription factors in zebrafish neural crest development and ectodermal evolution. J. Exp. Zool. B Mol. Dev. Evol..

[bib41] Hong C.S., Saint-Jeannet J.P. (2007). The activity of Pax3 and Zic1 regulates three distinct cell fates at the neural plate border. Mol. Biol. Cell.

[bib42] Huang D.W., Sherman B.T., Lempicki R.A. (2009). Bioinformatics enrichment tools: paths toward the comprehensive functional analysis of large gene lists. Nucleic Acids Res..

[bib43] Huang D.W., Sherman B.T., Lempicki R.A. (2009). Systematic and integrative analysis of large gene lists using DAVID bioinformatics resources. Nat. Protoc..

[bib44] Hwang C.H., Simeone A., Lai E., Wu D.K. (2009). Foxg1 is required for proper separation and formation of sensory cristae during inner ear development. Dev. Dynam..

[bib45] Jeon H., Waku T., Azami T., Khoa le T.P., Yanagisawa J., Takahashi S., Ema M. (2016). Comprehensive identification of kruppel-like factor family members contributing to the self-renewal of mouse embryonic stem cells and cellular reprogramming. PLoS One.

[bib46] Jiang L., Xu J., Jin R., Bai H., Zhang M., Yang S., Zhang X., Zhang X., Han Z., Zeng S. (2018). Transcriptomic analysis of chicken cochleae after gentamicin damage and the involvement of four signaling pathways (Notch, FGF, Wnt and BMP) in hair cell regeneration. Hear. Res..

[bib47] Kaji T., Artinger K.B. (2004). dlx3b and dlx4b function in the development of Rohon-Beard sensory neurons and trigeminal placode in the zebrafish neurula. Dev. Biol..

[bib48] Kajimura D., Dragomir C., Ramirez F., Laub F. (2007). Identification of genes regulated by transcription factor KLF7 in differentiating olfactory sensory neurons. Gene.

[bib49] Khan M.A.F., Soto-Jiminez L.M., Howe T., Streit A., Sosinsky A., Stern C.D. (2012). Computational tools and resources for prediction and analysis of gene regulatory regions in the chick genome. Genesis.

[bib50] Kharchenko P.V., Alekseyenko A.A., Schwartz Y.B., Minoda A., Riddle N.C., Ernst J., Sabo P.J., Larschan E., Gorchakov A.A., Gu T., Linder-Basso D., Plachetka A., Shanower G., Tolstorukov M.Y., Luquette L.J., Xi R., Jung Y.L., Park R.W., Bishop E.P., Canfield T.K., Sandstrom R., Thurman R.E., MacAlpine D.M., Stamatoyannopoulos J.A., Kellis M., Elgin S.C., Kuroda M.I., Pirrotta V., Karpen G.H., Park P.J. (2011). Comprehensive analysis of the chromatin landscape in Drosophila melanogaster. Nature.

[bib51] Khatri S.B., Edlund R.K., Groves A.K. (2014). Foxi3 is necessary for the induction of the chick otic placode in response to FGF signaling. Dev. Biol..

[bib52] Khatri S.B., Groves A.K. (2013). Expression of the Foxi2 and Foxi3 transcription factors during development of chicken sensory placodes and pharyngeal arches. Gene Expr. Patterns.

[bib53] Kim T.K., Hemberg M., Gray J.M., Costa A.M., Bear D.M., Wu J., Harmin D.A., Laptewicz M., Barbara-Haley K., Kuersten S., Markenscoff-Papadimitriou E., Kuhl D., Bito H., Worley P.F., Kreiman G., Greenberg M.E. (2010). Widespread transcription at neuronal activity-regulated enhancers. Nature.

[bib54] Kimura H. (2013). Histone modifications for human epigenome analysis. J. Hum. Genet..

[bib55] Knight R.D., Nair S., Nelson S.S., Afshar A., Javidan Y., Geisler R., Rauch G.J., Schilling T.F. (2003). Lockjaw encodes a zebrafish tfap2a required for early neural crest development. Development.

[bib56] Kniss J.S., Jiang L., Piotrowski T. (2016). Insights into sensory hair cell regeneration from the zebrafish lateral line. Curr. Opin. Genet. Dev..

[bib57] Kondoh H., Uchikawa M. (2008). Dissection of chick genomic regulatory regions. Methods Cell Biol..

[bib58] Ladher R.K. (2017). Changing shape and shaping change: inducing the inner ear. Semin. Cell Dev. Biol..

[bib59] Ladher R.K., Anakwe K.U., Gurney A.L., Schoenwolf G.C., Francis-West P.H. (2000). Identification of synergistic signals initiating inner ear development. Science.

[bib60] Laub F., Aldabe R., Friedrich V., Ohnishi S., Yoshida T., Ramirez F. (2001). Developmental expression of mouse Kruppel-like transcription factor KLF7 suggests a potential role in neurogenesis. Dev. Biol..

[bib61] Laub F., Dragomir C., Ramirez F. (2006). Mice without transcription factor KLF7 provide new insight into olfactory bulb development. Brain Res..

[bib62] Laub F., Lei L., Sumiyoshi H., Kajimura D., Dragomir C., Smaldone S., Puche A.C., Petros T.J., Mason C., Parada L.F., Ramirez F. (2005). Transcription factor KLF7 is important for neuronal morphogenesis in selected regions of the nervous system. Mol. Cell. Biol..

[bib63] Lee S.G., Huang M., Obholzer N.D., Sun S., Li W., Petrillo M., Dai P., Zhou Y., Cotanche D.A., Megason S.G., Li H., Chen Z.Y. (2016). Myc and fgf are required for zebrafish neuromast hair cell regeneration. PLoS One.

[bib64] Leger S., Brand M. (2002). Fgf8 and Fgf3 are required for zebrafish ear placode induction, maintenance and inner ear patterning. Mech. Dev..

[bib65] Li W., Cornell R.A. (2007). Redundant activities of Tfap2a and Tfap2c are required for neural crest induction and development of other non-neural ectoderm derivatives in zebrafish embryos. Dev. Biol..

[bib66] Litsiou A., Hanson S., Streit A. (2005). A balance of FGF, Wnt and BMP signalling positions the future placode territory in the head. Development.

[bib67] Long H.K., Prescott S.L., Wysocka J. (2016). Ever-changing landscapes: transcriptional enhancers in development and evolution. Cell.

[bib68] Luo T., Matsuo-Takasaki M., Sargent T.D. (2001). Distinct roles for Distal-less genes Dlx3 and Dlx5 in regulating ectodermal development in Xenopus. Mol. Reprod. Dev..

[bib69] Lush M.E., Diaz D.C., Koenecke N., Baek S., Boldt H., St Peter M.K., Gaitan-Escudero T., Romero-Carvajal A., Busch-Nentwich E.M., Perera A.G., Hall K.E., Peak A., Haug J.S., Piotrowski T. (2019). scRNA-Seq reveals distinct stem cell populations that drive hair cell regeneration after loss of Fgf and Notch signaling. Elife.

[bib70] Maier E.C., Whitfield T.T. (2014). RA and FGF signalling are required in the zebrafish otic vesicle to pattern and maintain ventral otic identities. PLoS Genet..

[bib71] Maroon H., Walshe J., Mahmood R., Kiefer P., Dickson C., Mason I. (2002). Fgf3 and Fgf8 are required together for formation of the otic placode and vesicle. Development.

[bib72] Matys V., Kel-Margoulis O.V., Fricke E., Liebich I., Land S., Barre-Dirrie A., Reuter I., Chekmenev D., Krull M., Hornischer K., Voss N., Stegmaier P., Lewicki-Potapov B., Saxel H., Kel A.E., Wingender E. (2006). TRANSFAC and its module TRANSCompel: transcriptional gene regulation in eukaryotes. Nucleic Acids Res..

[bib73] McLarren K.W., Litsiou A., Streit A. (2003). DLX5 positions the neural crest and preplacode region at the border of the neural plate. Dev. Biol..

[bib74] Miotto B., Sagnier T., Berenger H., Bohmann D., Pradel J., Graba Y. (2006). Chameau HAT and DRpd3 HDAC function as antagonistic cofactors of JNK/AP-1-dependent transcription during Drosophila metamorphosis. Genes Dev..

[bib75] Mittelstadt M.L., Patel R.C. (2012). AP-1 mediated transcriptional repression of matrix metalloproteinase-9 by recruitment of histone deacetylase 1 in response to interferon beta. PLoS One.

[bib76] Mueller K.L., Jacques B.E., Kelley M.W. (2002). Fibroblast growth factor signaling regulates pillar cell development in the organ of corti. J. Neurosci..

[bib77] Neuberg M., Schuermann M., Hunter J.B., Muller R. (1989). Two functionally different regions in Fos are required for the sequence-specific DNA interaction of the Fos/Jun protein complex. Nature.

[bib78] Nicol J.W., Helt G.A., Blanchard S.G., Raja A., Loraine A.E. (2009). The Integrated Genome Browser: free software for distribution and exploration of genome-scale datasets. Bioinformatics.

[bib79] O’Donnell A., Yang S.H., Sharrocks A.D. (2008). MAP kinase-mediated c-fos regulation relies on a histone acetylation relay switch. Mol. Cell.

[bib80] Ohyama T., Groves A.K., Martin K. (2007). The first steps towards hearing: mechanisms of otic placode induction. Int. J. Dev. Biol..

[bib81] Ohyama T., Mohamed O.A., Taketo M.M., Dufort D., Groves A.K. (2006). Wnt signals mediate a fate decision between otic placode and epidermis. Development.

[bib82] Ornitz D.M., Itoh N. (2015). The fibroblast growth factor signaling pathway. Wiley Interdiscip. Rev. Dev. Biol..

[bib83] Pandur P.D., Moody S.A. (2000). Xenopus Six1 gene is expressed in neurogenic cranial placodes and maintained in the differentiating lateral lines. Mech. Dev..

[bib84] Park B.Y., Saint-Jeannet J.P. (2008). Hindbrain-derived Wnt and Fgf signals cooperate to specify the otic placode in Xenopus. Dev. Biol..

[bib85] Patel N.S., Rhinn M., Semprich C.I., Halley P.A., Dolle P., Bickmore W.A., Storey K.G. (2013). FGF signalling regulates chromatin organisation during neural differentiation via mechanisms that can be uncoupled from transcription. PLoS Genet..

[bib86] Paten B., Herrero J., Beal K., Fitzgerald S., Birney E. (2008). Enredo and Pecan: genome-wide mammalian consistency-based multiple alignment with paralogs. Genome Res..

[bib87] Pennacchio L.A., Bickmore W., Dean A., Nobrega M.A., Bejerano G. (2013). Enhancers: five essential questions. Nat. Rev. Genet..

[bib88] Phillips B.T., Bolding K., Riley B.B. (2001). Zebrafish fgf3 and fgf8 encode redundant functions required for otic placode induction. Dev. Biol..

[bib89] Qiao Y., Zhu Y., Sheng N., Chen J., Tao R., Zhu Q., Zhang T., Qian C., Jing N. (2012). AP2[gamma] regulates neural and epidermal development downstream of the BMP pathway at early stages of ectodermal patterning. Cell Res..

[bib90] Rada-Iglesias A., Bajpai R., Prescott S., Brugmann S.A., Swigut T., Wysocka J. (2012). Epigenomic annotation of enhancers predicts transcriptional regulators of human neural crest. Cell Stem Cell.

[bib91] Rada-Iglesias A., Bajpai R., Swigut T., Brugmann S.A., Flynn R.A., Wysocka J. (2011). A unique chromatin signature uncovers early developmental enhancers in humans. Nature.

[bib92] Saint-Jeannet J.P., Moody S.A. (2014). Establishing the pre-placodal region and breaking it into placodes with distinct identities. Dev. Biol..

[bib93] Sandelin A., Alkema W., Engstrom P., Wasserman W.W., Lenhard B. (2004). JASPAR: an open-access database for eukaryotic transcription factor binding profiles. Nucleic Acids Res..

[bib94] Sato S., Ikeda K., Shioi G., Ochi H., Ogino H., Yajima H., Kawakami K. (2010). Conserved expression of mouse Six1 in the pre-placodal region (PPR) and identification of an enhancer for the rostral PPR. Dev. Biol..

[bib95] Schlosser G. (2014). Early embryonic specification of vertebrate cranial placodes. Wiley Interdiscip. Rev. Dev. Biol..

[bib96] Semprich C.I., Metzis V., Patel H., Briscoe J., Storey K.G. (2019). ERK1/2 Signalling Dynamics Promote Neural Differentiation by Regulating the Polycomb Repressive Complex.

[bib97] Shim K., Minowada G., Coling D.E., Martin G.R. (2005). Sprouty2, a mouse deafness gene, regulates cell fate decisions in the auditory sensory epithelium by antagonizing FGF signaling. Dev. Cell.

[bib98] Shlyueva D., Stampfel G., Stark A. (2014). Transcriptional enhancers: from properties to genome-wide predictions. Nat. Rev. Genet..

[bib99] Soloaga A., Thomson S., Wiggin G.R., Rampersaud N., Dyson M.H., Hazzalin C.A., Mahadevan L.C., Arthur J.S. (2003). MSK2 and MSK1 mediate the mitogen- and stress-induced phosphorylation of histone H3 and HMG-14. EMBO J..

[bib100] Solomon K.S., Kudoh T., Dawid I.B., Fritz A. (2003). Zebrafish foxi1 mediates otic placode formation and jaw development. Development.

[bib101] Streit A., Watt F., Gage F. (2008). The cranial sensory nervous system: specification of sensory progenitors and placodes. StemBook, 2010/07/09.

[bib102] Streit A. (2018). Specification of sensory placode progenitors: signals and transcription factor networks. Int. J. Dev. Biol..

[bib103] Streit A., Stern C.D. (2001). Combined whole-mount in situ hybridization and immunohistochemistry in avian embryos. Methods.

[bib104] Sun S.K., Dee C.T., Tripathi V.B., Rengifo A., Hirst C.S., Scotting P.J. (2007). Epibranchial and otic placodes are induced by a common Fgf signal, but their subsequent development is independent. Dev. Biol..

[bib105] Thomas-Chollier M., Darbo E., Herrmann C., Defrance M., Thieffry D., van Helden J. (2012). A complete workflow for the analysis of full-size ChIP-seq (and similar) data sets using peak-motifs. Nat. Protoc..

[bib106] Thomas-Chollier M., Herrmann C., Defrance M., Sand O., Thieffry D., van Helden J. (2012). RSAT peak-motifs: motif analysis in full-size ChIP-seq datasets. Nucleic Acids Res..

[bib107] Tsang M., Dawid I.B. (2004). Promotion and attenuation of FGF signaling through the Ras-MAPK pathway. Sci. STKE.

[bib108] Turatsinze J.V., Thomas-Chollier M., Defrance M., van Helden J. (2008). Using RSAT to scan genome sequences for transcription factor binding sites and cis-regulatory modules. Nat. Protoc..

[bib109] Urness L.D., Paxton C.N., Wang X., Schoenwolf G.C., Mansour S.L. (2010). FGF signaling regulates otic placode induction and refinement by controlling both ectodermal target genes and hindbrain Wnt8a. Dev. Biol..

[bib110] Vierbuchen T., Ling E., Cowley C.J., Couch C.H., Wang X.F., Harmin D.A., Roberts C.W.M., Greenberg M.E. (2017). AP-1 transcription factors and the BAF complex mediate signal-dependent enhancer selection. Mol. Cell.

[bib111] Visel A., Blow M.J., Li Z., Zhang T., Akiyama J.A., Holt A., Plajzer-Frick I., Shoukry M., Wright C., Chen F., Afzal V., Ren B., Rubin E.M., Pennacchio L.A. (2009). ChIP-seq accurately predicts tissue-specific activity of enhancers. Nature.

[bib112] Wang D., Garcia-Bassets I., Benner C., Li W., Su X., Zhou Y., Qiu J., Liu W., Kaikkonen M.U., Ohgi K.A., Glass C.K., Rosenfeld M.G., Fu X.D. (2011). Reprogramming transcription by distinct classes of enhancers functionally defined by eRNA. Nature.

[bib113] Whitfield T.T. (2015). Development of the inner ear. Curr. Opin. Genet. Dev..

[bib114] Whitfield T.T., Riley B.B., Chiang M.Y., Phillips B. (2002). Development of the zebrafish inner ear. Dev. Dynam..

[bib115] Wright T.J., Mansour S.L. (2003). Fgf3 and Fgf10 are required for mouse otic placode induction. Development.

[bib116] Yang L., O’Neill P., Martin K., Maass J.C., Vassilev V., Ladher R., Groves A.K. (2013). Analysis of FGF-dependent and FGF-independent pathways in otic placode induction. PLoS One.

[bib117] Yang S.H., Sharrocks A.D., Whitmarsh A.J. (2003). Transcriptional regulation by the MAP kinase signaling cascades. Gene.

[bib118] Yang S.H., Sharrocks A.D., Whitmarsh A.J. (2013). MAP kinase signalling cascades and transcriptional regulation. Gene.

[bib119] Zentner G.E., Tesar P.J., Scacheri P.C. (2011). Epigenetic signatures distinguish multiple classes of enhancers with distinct cellular functions. Genome Res..

[bib120] Zhang Y., Liu T., Meyer C.A., Eeckhoute J., Johnson D.S., Bernstein B.E., Nussbaum C., Myers R.M., Brown M., Li W., Liu X.S. (2008). Model-based analysis of ChIP-seq (MACS). Genome Biol..

[bib121] Zhu L.J., Gazin C., Lawson N.D., Pages H., Lin S.M., Lapointe D.S., Green M.R. (2010). ChIPpeakAnno: a bioconductor package to annotate ChIP-seq and ChIP-chip data. BMC Bioinf..

