# Supplementary material for: Enhancer activation by FGF signalling during otic induction
Source: Dev Biol. 2020 Jan 1;457(1):69–82. doi: 10.1016/j.ydbio.2019.09.006 (PMC6902270; doi:10.1016/j.ydbio.2019.09.006)
Supplement: Multimedia component 4 [file mmc4.docx]

**
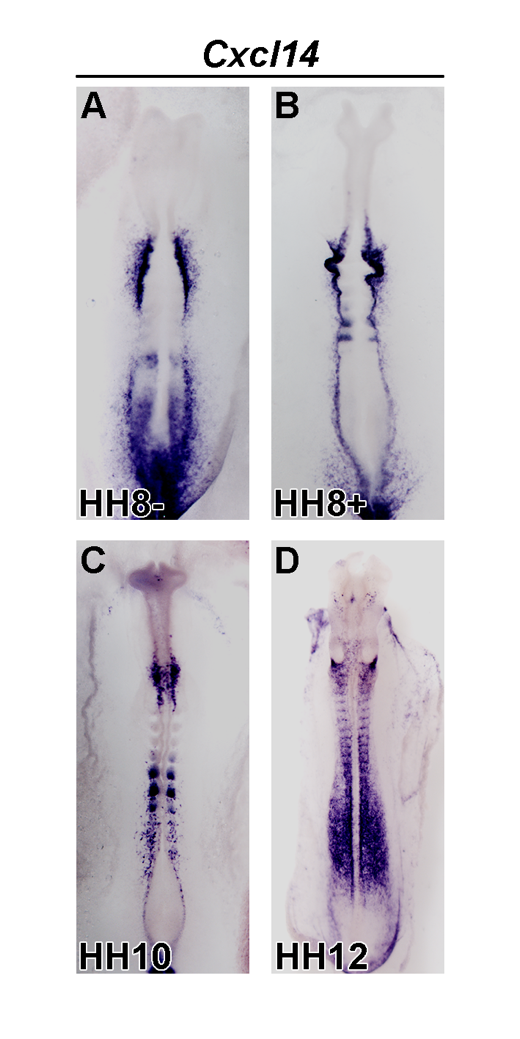
**

**Supplementary Figure 1. Expression pattern of *Cxlc14*.**

*In situ* hybridisation showing the expression of *Cxcl14* in chick embryos at the 3- (A), 5- (B), 10- (C) and 15- (D) somite stage. *Cxcl14* becomes strongly expressed in the neural folds and the ectoderm just lateral to them in the region where OEPs are induced, and is also expressed in the ectoderm along border of the posterior neural plate and in somites. At otic level, its expression first seems to narrow restricting towards the neural tube (C) and then broadens in the ectoderm to surround the otic placode (D).


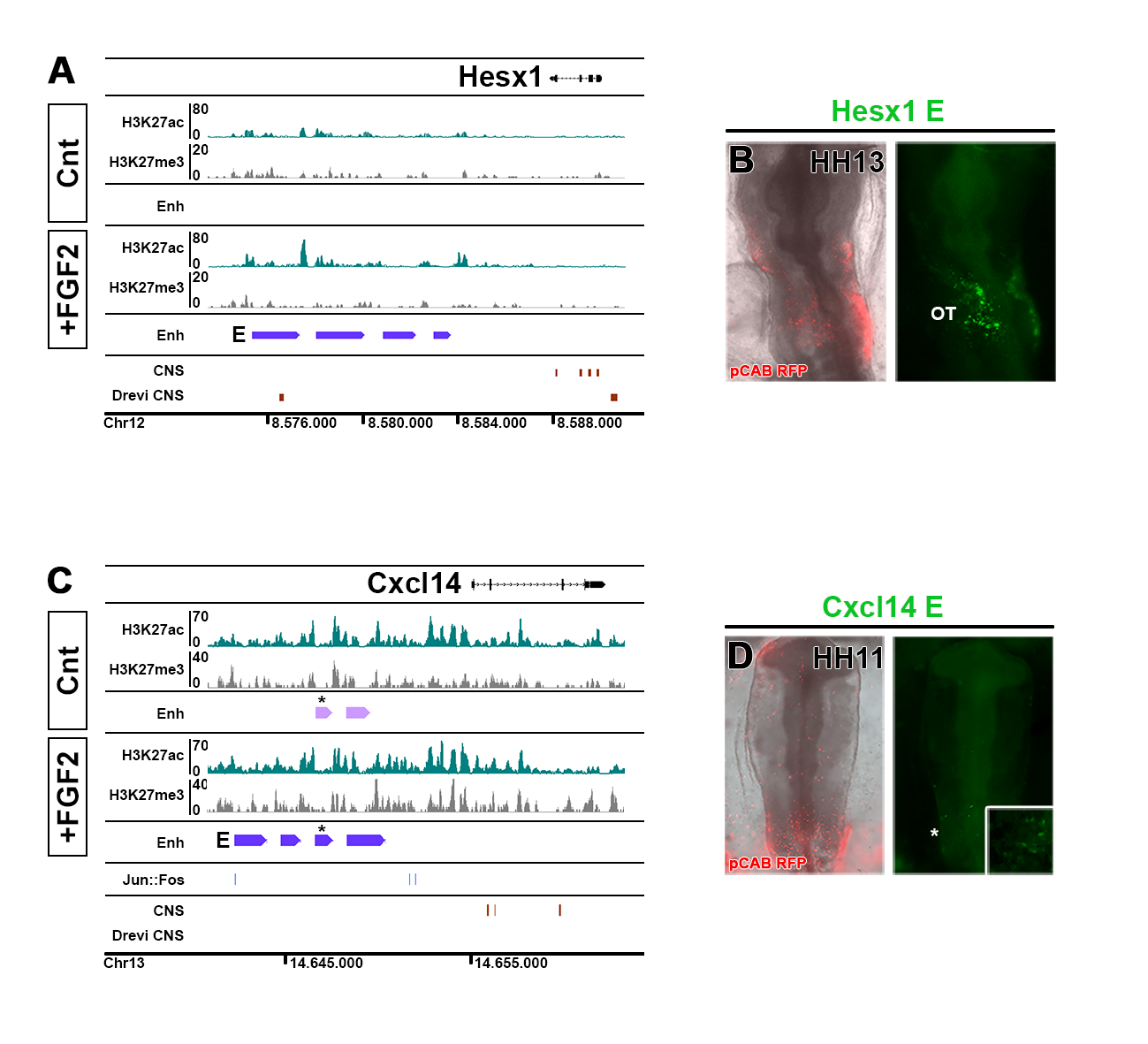


**Supplementary Figure 2. Hesx1 and Cxcl14 enhancers.**

IGB browser view of Hesx1 (A) and Cxcl14 (C) enhancers. ChIP-identified enhancers are in violet for +FGF2 and pink for control samples (* marks common enhancers between Cnt and +FGF2); H3K27ac track is shown in green and H3K27me3 in grey. Jun::Fos putative binding sites are shown for Cxcl14 locus (blue); conserved non-coding sequences (CNS) are in red. Characterization of *in vivo* activity of Hesx1 E (green channel) shows that it is active in the otic placode (OT) at HH13 (B). The Cxcl14 enhancer element is active in few cells in the neural tube and in the ectoderm surrounding the otic placode (magnified inset) (D).


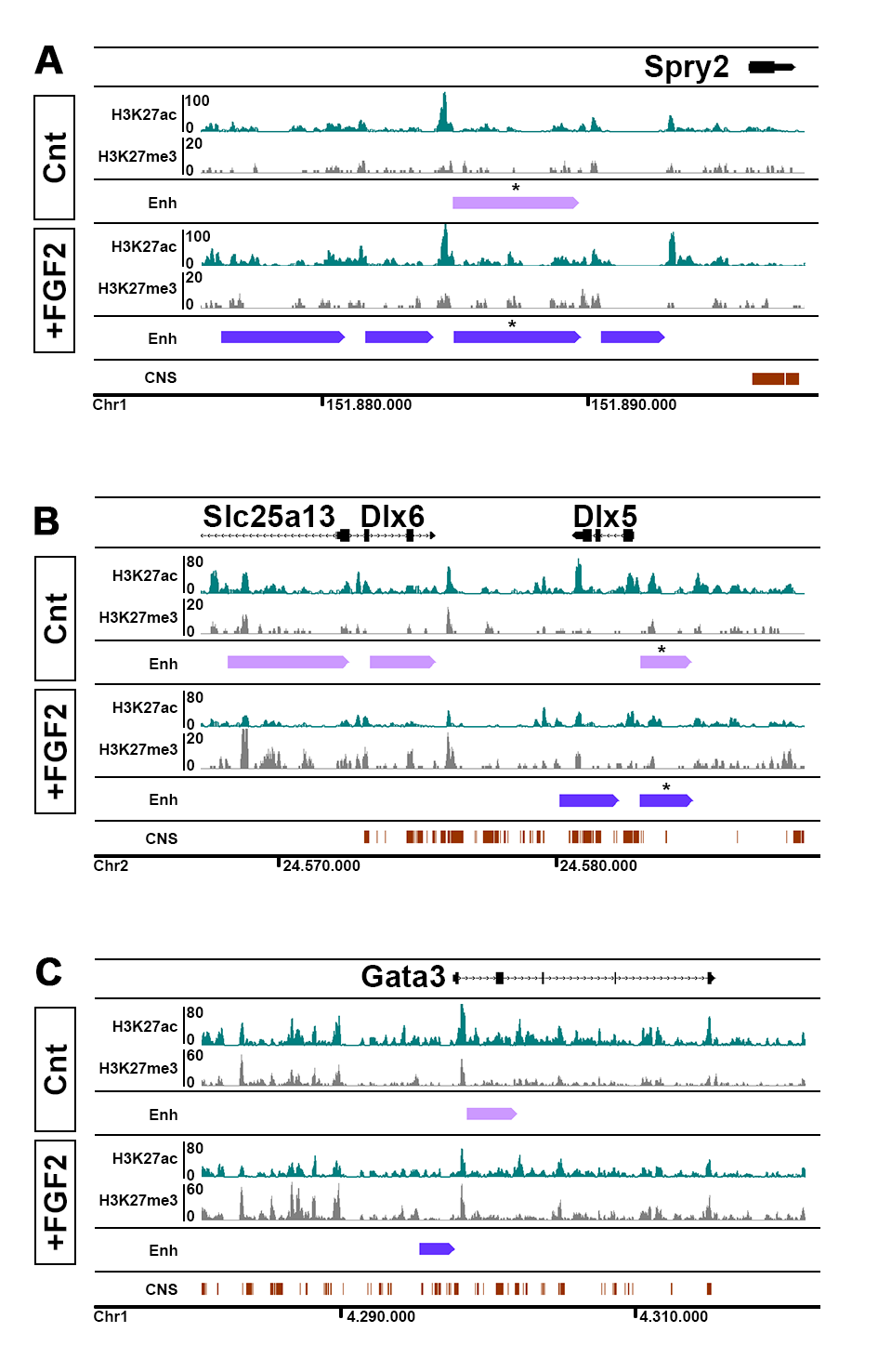


**Supplementary Figure 3. Genomic regions surrounding Spry2, Dlx5/6 and Gata3.**

IGB browser view of the Spry2 (A), Dlx5/6 (B) and Gata3 (C) locus. ChIP-identified enhancers are in violet for +FGF2 and pink for control samples (* marks common enhancers between Cnt and +FGF2); H3K27ac track is shown in green and H3K27me3 in grey. Conserved non-coding sequences (CNS) are in red. FGF2 induction increases H3K27ac around Spry2 (A) while there is a decrease in H3K27ac and a gain in H3K27me3 in Dlx5/6 (B) and Gata3 (C), which are genes negatively regulated by FGF signalling.


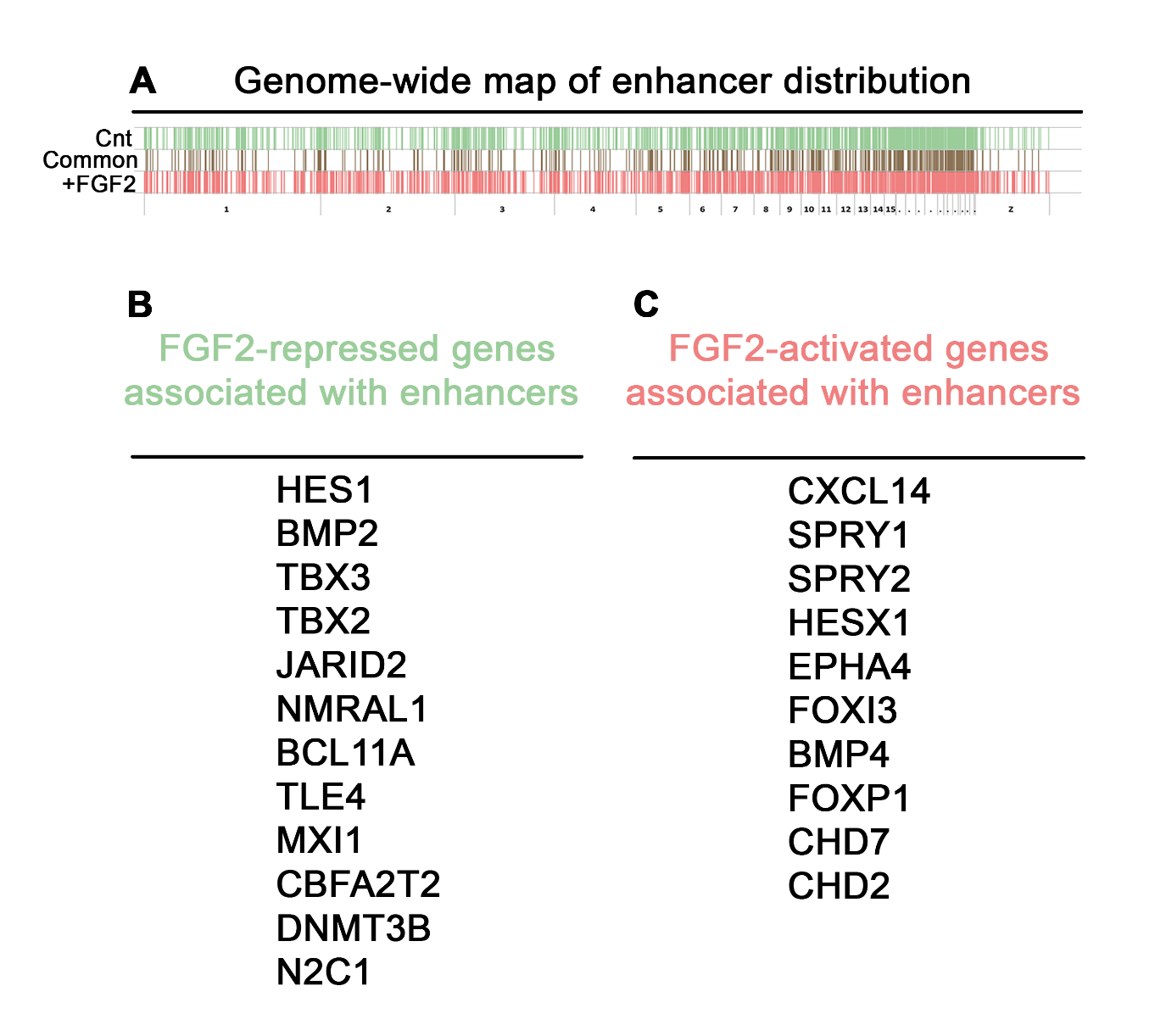


**Supplementary Figure 4. Genome-wide map of enhancers identified in control and +FGF2 treated sensory progenitors.**

(A) A genome-wide view of the location of common and unique enhancers in +FGF2 and control samples. (B) List of genes significantly downregulated by FGF2 with an associated proximal enhancer determined by ChIP-seq in control sensory progenitors. (C) List of genes significantly upregulated by FGF2 with an associated proximal enhancer determined by ChIP-seq in FGF2-treated sensory progenitors.


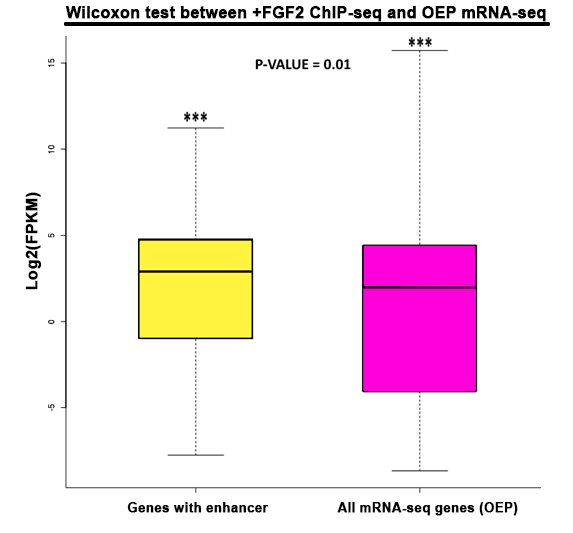


**Supplementary Figure 5. Wilcoxon test reveals a significant correlation between +FGF2 ChIP-seq and OEP mRNA-seq.**

Putative enhancers in FGF2 treated sensory progenitors were defined as maximum 3 kb genomic regions flanked by H3K27ac peaks and devoid of H3K27me3 peaks. These were annotated to the nearest TSS. mRNA-seq for OEPs were retrieved from Chen and colleagues (Chen et al., 2017). A Wilcoxon test was carried out to test if the mean FPKM of genes with putative enhancers in FGF2 treated sensory progenitors is greater than the mean FPKM of all OEP genes. This analysis shows that indeed enhancer associated genes have high expression levels in OEPs with a significant p-value of 0.01.


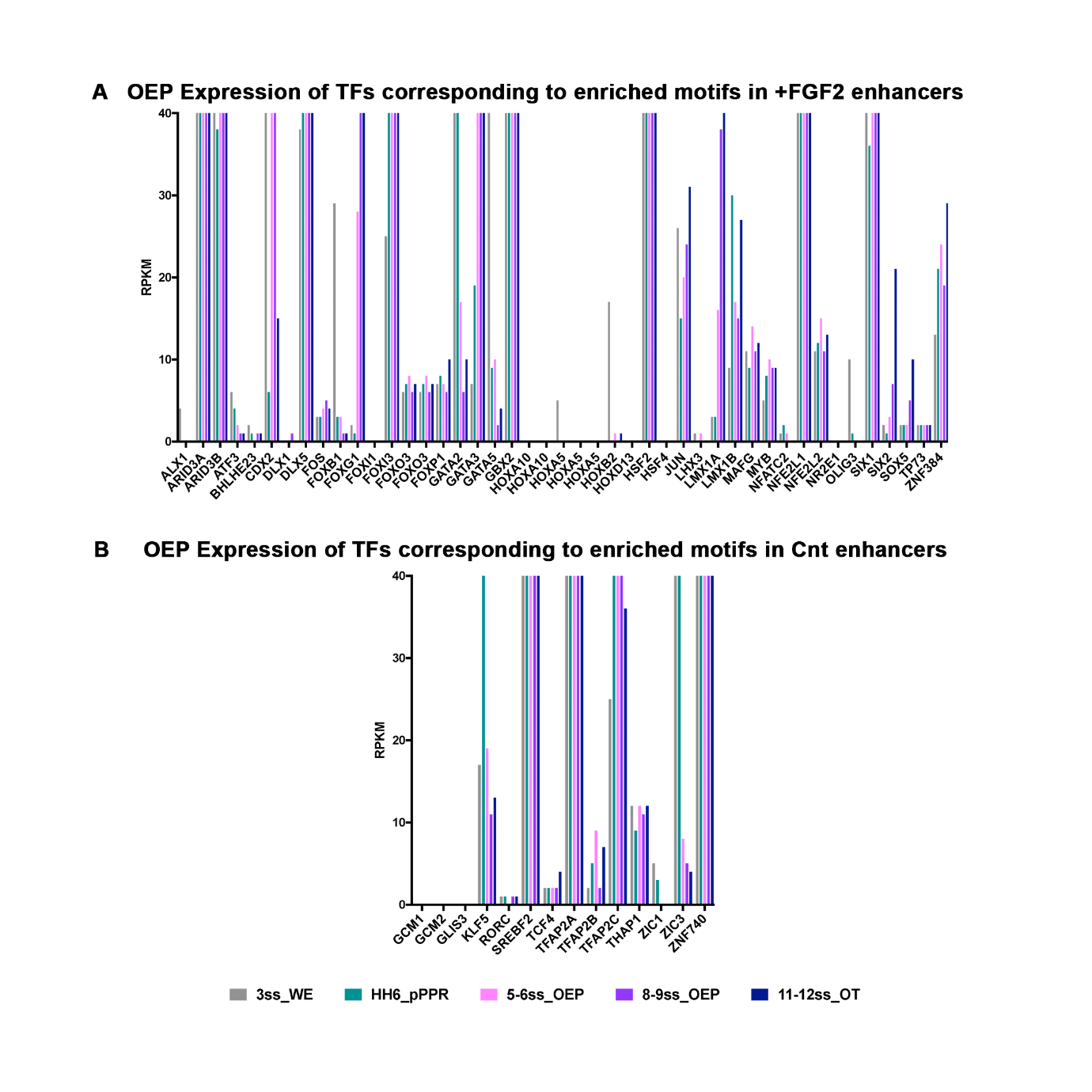


**Supplementary Figure 6. Otic expression of transcription factors corresponding to enriched enhancer motifs.**

Expression level in 3ss whole embryo (grey), 0ss HH6 pPPR (green), 5-6ss OEP (pink), 8-9ss OEP (violet) and 11-12ss otic placode (blue) from Chen and colleagues (Chen et al., 2017) was plotted for transcription factors corresponding to enriched motifs in +FGF2 (A) and control (B) enhancers.


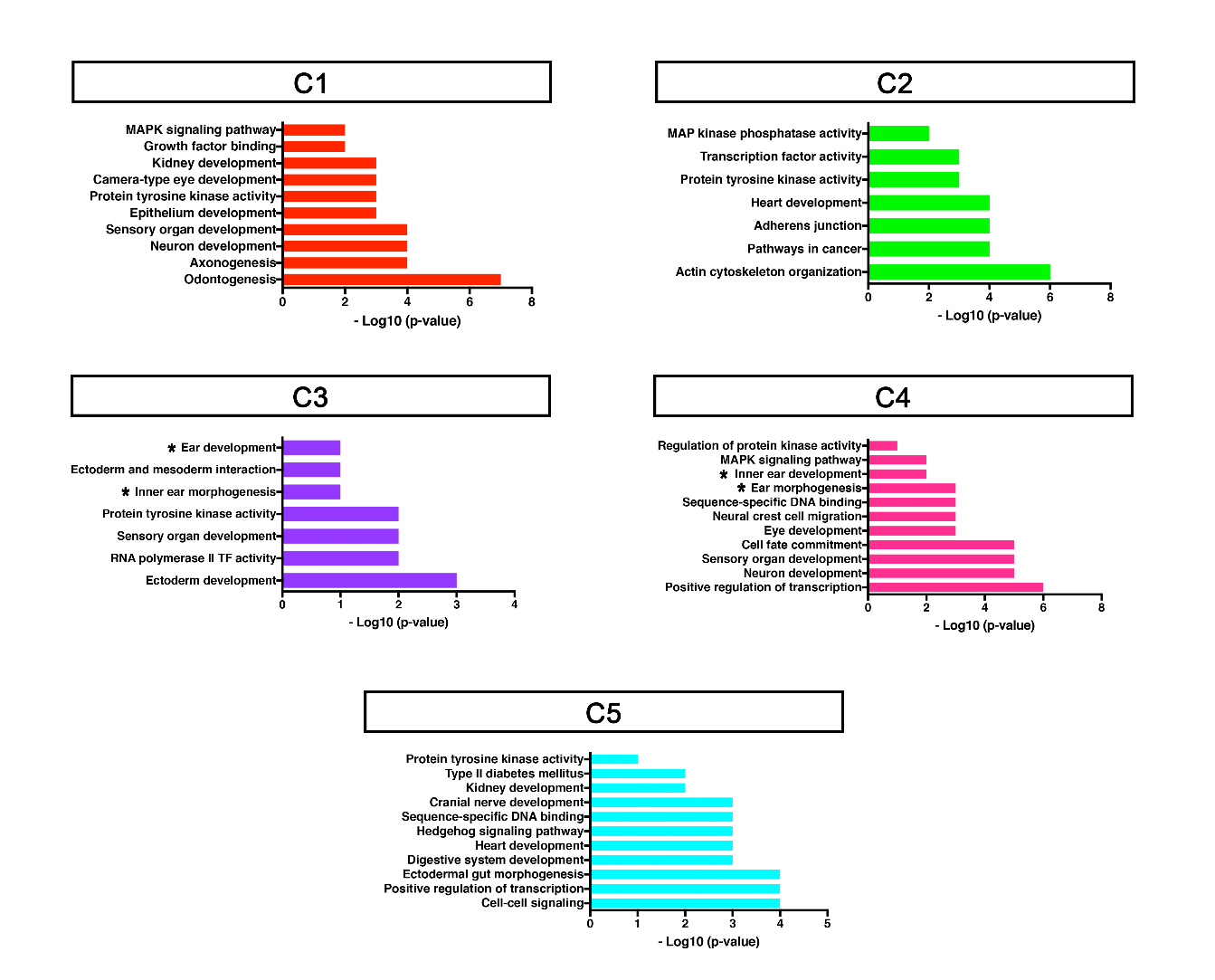


**Supplementary Figure 7. Gene ontology for genes associated with dense H3K27ac peaks flanking Ap1 binding sites.**

To assess each cluster Gene Ontology (GO) and KEGG terms were identified using DAVID. GO terms are coloured according to the colours of each cluster in Figure 3. Cluster 3 and 4 contain putative enhancers for some of the earliest FGF-target genes during OEP induction hence the enriched terms: inner ear morphogenesis and MAPK signalling.

**Supplementary Methods**

**Supplementary Table 1. Enhancer cloning Primers**

| Enhancer Cloning Primers | | |
| --- | --- | --- |
| Spry1 E | F 5’-ACGCCTCTCTACCCTCTTT-3’  R 5’-GCTGGAAGCTAGAGCCATATC-3’ | Chr4: 52768022 - 52768515  494bp |
| Foxi3 E1 | F 5’-TCTGACATTTCATCATGGCTTCA-3’  R 5’-GGTCATCTGAATGACAACTGTCTC-3’ | Chr4: 85595147- 85595756  610bp |
| Foxi3 E2 | F 5’-TTTGGCCCTGTTCAAATGG-3’  R 5’-CAGTTTGTTGATACCTTCAGTGT-3’ | Chr4: 85611260 - 85611770  511bp |
| Hesx1 E | F 5’-CAACTGCTTTCTATAATGTGTACCAG-3’  R 5’-GCGTTTGATTATCGTGCTGTC-3’ | Chr12: 8576366 -  8576880  515bp |
| Cxcl14 E | F 5’-AGCCTACCAGTTGTCCTAGA-3’  R 5’-CACAGTGTATTGCTTGGCTTT-3’ | Chr13: 14642196 -14643846  1651bp |

**Supplementary Table 2. Mutagenesis Primers**

| Mutagenesis Primers | | |
| --- | --- | --- |
| Foxi3 E1  ΔSoxE | F 5’-TCTGACATTTCATCATGGCTTCA-3’  R 5’-TTGCAAAAGGAAAAGAAGCAGG-3’ | Tag3 |
| Foxi3 E1  ΔSoxD | **(P1)** F 5’-TGAAGCCATGATGAAATGTCAGA-3’  **(P2)** R 5’-CATCCCTGCAGGCTATTTGGCACTGTGATGCAGTG-3’  **(P3)** F 5’-CACTGCATCACAGTGCCAAATAGCCTGCAGGGATG-3’  **(P4)** R 5’-GAGACAGTTGTCATTCAGATGACC-3’ | Tag4 |
| Foxi3 E1  ΔTead1 | **(P1)** F 5’-TGAAGCCATGATGAAATGTCAGA -3’  **(P2)** R 5’-GAACATAAGTATAAATTTCTTCACATCAACACTGAAGTTAAGCA-3’  **(P3)** F 5’-TGCTTAACTTCAGTGTTGATGTGAAGAAATTTATACTTATGTTC-3’  **(P4)** R 5’-GAGACAGTTGTCATTCAGATGACC-3’ | Tag5 |
| Foxi3 E1  ΔSoxE/D | **(P1)** F 5’-GGACTAGTTCTGACATTTCATCATGGCTTCA -3’  **(P2)** R 5’-CATCCCTGCAGGCTATTTGGCACTGTGATGCAGTG-3’  **(P3)** F 5’-CACTGCATCACAGTGCCAAATAGCCTGCAGGGATG-3’  **(P4)** R 5’-TTGCAAAAGGAAAAGAAGCAGG-3’ | Tag8 |

**Supplementary Table 3. ChIP-qPCR Primers**

| P300-Flag ChIP-qPCR Primers | |
| --- | --- |
| Spry1 E | F 5’-CCTCTATCCCTTTGGTTGTACG-3’  R 5’-GATAATGTTTGCTCTGCGGTTC-3’ |
| Foxi3 E1 | F 5’-GCAGGGATGGCCTTACATCA-3’  R 5’-ACGTGCAGCCATGGAACATA-3’ |
| MyoD N | F 5’-AGTCACCTCCACCTAAAATGC-3’  R 5’-TGCATGACCGAAGTGTAAGG-3’ |

**Supplementary File 1. Nanostring**

**A_3h FGF_Analysis:** Normalised gene level of expression is reported for each of the three replicates of Cnt and +FGF2 3h treatment. Gene are sorted based on fold change and p-value. Significant upregulated genes (FC>=1.25; pvalue<=0.05) and downregulated genes (FC>=0.75; pvalue<=0.05) have been annotated with gene function and expression domain.

**B_3h CHX_Analysis:** Normalised gene level of expression is reported for each of the three replicates of Cnt DMSO and +FGF2 +CHX 3h treatment. Gene are sorted based on fold change and p-value. Significant upregulated genes (FC>=1.25; pvalue<=0.05) and downregulated genes (FC>=0.75; pvalue<=0.05) have been annotated with gene function and expression domain.

**C_3h CHX vs 3h_Analysis:** Common genes upregulated or downregulated at 3h (A) and in the 3h CHX experiment (B) have been listed here. Note: these are considered to be FGF direct targets.

**D_6h FGF_Analysis:** Normalised gene level of expression is reported for each of the three replicates of Cnt and +FGF2 6h treatment. Gene are sorted based on fold change and p-value. Significant upregulated genes (FC>=1.25; pvalue<=0.05) and downregulated genes (FC>=0.75; pvalue<=0.05) have been annotated with gene function and expression domain.

**Supplementary File 2. Cnt_TFBS_Motif_Enrichment**

Summary of RSAT motif enrichment analysis in Cnt enhancer with +FGF2 enhancers as background. Each excel sheet summarises the identified transcription factors for each matrix (Cnt_M1 to Cnt_M30). When no known transcription factor was associated with the enriched motif only k-mer and evalue were reported.

**Supplementary File 3. FGF2_TFBS_Motif_Enrichment**

Summary of RSAT motif enrichment analysis in +FGF2 enhancer with Cnt enhancers as background. Each excel sheet summarises the identified transcription factors for each matrix (Cnt_M1 to Cnt_M30). When no known transcription factor was associated with the enriched motif only k-mer and evalue were reported.
